# Comparison of Brain Activity Correlating with Self-Report versus Narrative Attachment Measures during Conscious Appraisal of an Attachment Figure

**DOI:** 10.3389/fnhum.2016.00090

**Published:** 2016-03-14

**Authors:** Zimri S. Yaseen, Xian Zhang, J. Christopher Muran, Arnold Winston, Igor I. Galynker

**Affiliations:** ^1^Department of Psychiatry, Mount Sinai Beth IsraelNew York, NY, USA; ^2^Department of Psychiatry, Yale School of MedicineNew Haven, CT, USA; ^3^Department of Psychology, Derner Institute, Adelphi UniversityGarden City, NY, USA

**Keywords:** attachment, attachment measures, adult attachment, AAI, RSQ, fMRI

## Abstract

**Objectives:** The Adult Attachment Interview (AAI) has been the gold standard of attachment assessment, but requires special training. The Relationship Scales Questionnaire (RSQ) is a widely used self-report measure. We investigate how each correlates with brain activity during appraisal of subjects’ mothers.

**Methods:** Twenty-eight women were scored on the AAI, RSQ, and mood measures. During functional magnetic resonance imaging, subjects viewed their mothers in neutral-, valence-, and salience-rating conditions. We identified regions where contrasts in brain activity between appraisal and neutral viewing conditions correlated with each measure of attachment after covarying for mood. AAI and RSQ measures were then compared in terms of the extent to which regions of correlating brain activity overlapped with “default mode network” (DMN) vs. executive frontal network (EFN) masks and cortical vs. subcortical masks. Additionally, interactions with mood were examined.

**Results:** Salience and valence processing associated with increased thalamo-striatal, posterior cingulate, and visual cortex activity. Salience processing decreased PFC activity, whereas valence processing increased left insula activity. Activity correlating with AAI vs. RSQ measures demonstrated significantly more DMN and subcortical involvement. Interactions with mood were observed in the middle temporal gyrus and precuneus for both measures.

**Conclusion:** The AAI appears to disproportionately correlate with conscious appraisal associated activity in DMN and subcortical structures, while the RSQ appears to tap EFN structures more extensively. Thus, the AAI may assess more interoceptive, ‘core-self’-related processes, while the RSQ captures higher-order cognitions involved in attachment. Shared interaction effects between mood and AAI and RSQ-measures may suggest that processes tapped by each belong to a common system.

## Introduction

### Attachment and its Role in Psychotherapy Research

Measuring patient changes has been a longstanding challenge for psychotherapy outcome research, and patient personality in addition to symptomatology and functioning is an important consideration in the assessment of patient change (Strupp et al., 1997). Since the publication of Strupp et al.’s (1997) volume on assessment of patient change in psychotherapy, there have been many significant contributions to the domain of personality measurement in the context of psychotherapy research. Recently, Dimaggio et al. (2013) organized a special issue for *Psychotherapy Research* in which this subject matter received considerable attention. In this issue, they once again drew attention to attachment theory as a valuable approach to understanding personality. Attachment characteristics may themselves be important treatment targets (Taylor et al., 2015). Change in attachment characteristics may be a particularly important outcome in therapies aimed at long-term change in personality functioning (for example, transference and schema focused therapies; Giesen-Bloo et al., 2006; Levy et al., 2006; Doering et al., 2010). Attachment characteristics are thus important personality traits that bear on the assessment and understanding of treatment efficacy as direct measures thereof, but also in numerous other ways. Studies examining attachment characteristics’ bearing on psychotherapy include investigations of: (1) patient factors in treatment efficacy (Wei et al., 2004), (2) patient factors in treatment process (Mallinckrodt, 2000; Mallinckrodt et al., 2005), (3) therapist factors in treatment efficacy (Bruck et al., 2006), (4) therapist factors in treatment process and alliance formation (Sauer et al., 2003), and (5) patient-therapist matching (Wiseman and Tishby, 2014).

For research to proceed in these domains, understanding measures of attachment is imperative. Furthermore, as neuroscience becomes increasingly able to inform psychotherapy research (Weingarten and Strauman, 2015), and even psychotherapy design (Alexopoulos and Arean, 2014), an understanding of the neural activity correlates of specific attachment measures (and ultimately the neural bases of their meaning) during psychotherapy relevant tasks such as attachment figure appraisal is necessary if this fundamental domain is to be included in the conversation.

### The Constructs of Attachment and Their Neural Bases

**FIGURE 1:**
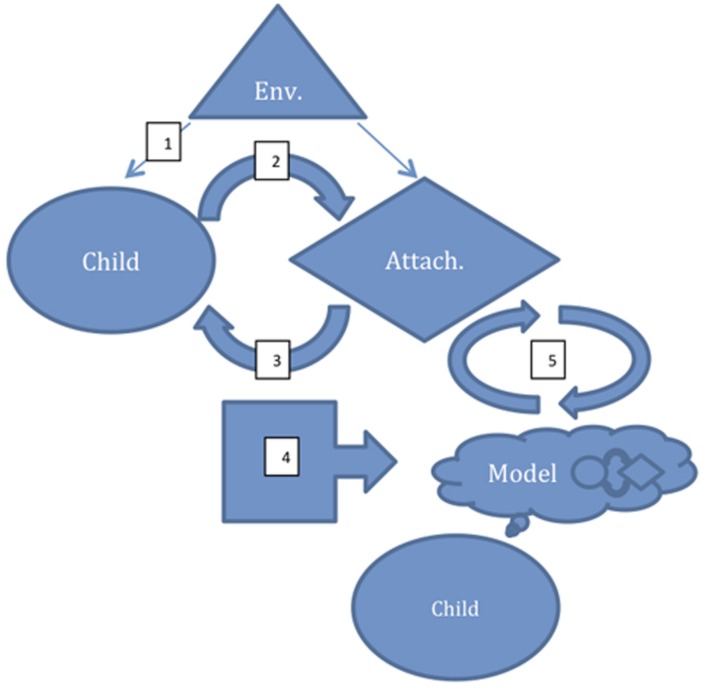
**A schematic model of the development of an internal working model for attachment.** (1) Perception of an external stimulus as one that should be approached or avoided. (2) Affective/behavioral response to stimulus (influencing the attachment figure). (3) Perception of attachment figure response to the environmental stimulus-affective/behavioral response pair. (4) Recursion of the cycle resulting in modeling/prediction of the attachment figure’s responses to such affect/behavior. (5) Generation of a behavioral responses matching the model with actual attachment figure response as the initiating external stimulus; comparison of predicted and actual response refines model. Env, environmental stimulus; Attach, Attachment figure.

Attachment characteristics are thought to be relatively stable traits that develop in infancy, arising from the patterns of interaction between the infant and its caregivers (typically most importantly the mother), who serve as primary attachment figures. They describe a working model of others and their response to one’s distress that in turn guides one’s own patterns of behavior in response to distress (Wallin, 2007). The development of attachment is, therefore, a complex phenomenon that combines in a programmatic and cyclical way exteroceptive processes (oriented to perception and processing of external stimuli–in particular, detection of goals, threats, and attachment figures’ affective responses), and interoceptive processes (oriented to perception and processing of internal states–in particular core affect generation, regulation, and recognition), to modulate responses to environmental stimuli according to attachment figure responses (Vrticka and Vuilleumier, 2012). (see **Figure 1**).

Ultimately, this iterative process should, on the neural level, involve networks that function to evaluate basic affects as well as those that are involved in mental state representations and affect regulation (Vrticka and Vuilleumier, 2012). Indeed, recent work has emphasized and begun to clarify the structures of interacting distributed brain networks whose activity generates mental experience (Pessoa, 2008; Spreng et al., 2013). Such work has emerged from a basic division between anticorrelated networks of brain activity: the primarily lateral, primarily exteroceptive task-positive networks, active during task performance, and the primarily medial, primarily interoceptive task negative network, active when individuals are not engaged in a particular task (Fox et al., 2005). Moreover, as recent work by Dr. Ochsner’s group on elements of affective response shows, some of the integration of medial cortical structures in cognitive control (task-positive) networks may be a function of necessary involvement of interoception in many cognitive control tasks (Satpute et al., 2013).

As the development of attachment results from recursive interplay between interoceptive and exteroceptive processes, it might be expected that neural activity that is involved in tasks pertinent to attachment representations and that varies with attachment characteristics (i.e., correlates with attachment measures) should link multiple interacting networks at different levels (Vrticka and Vuilleumier, 2012). In particular, networks involved in the generation of basic affects [most directly, the ‘PANIC’ system, described by Panksepp, responsible for separation distress (Panksepp, 2005)] and valence systems (Lindquist et al., 2015), as well as networks involved in tasks of social cognition such as recognition of others’ affects and empathy (Bernhardt and Singer, 2012) should be involved in attachment processes. Finally the activity patterns of affect-regulating circuits linking cortical regulatory structures to subcortical and cortical affect generating structures in negative feedback loops may vary with attachment characteristics. Indirect evidence for this is provided, for example, by Borchardt et al. (2015) study of resting state network connectivity pattern changes in response to narratives characteristic of different prototypical attachment representations; there they found decreased network connectivity for the supplemental motor area (implicated in empathic processes) following attention to biographical narratives presented in a dismissing attachment manner (Borchardt et al., 2015).

The mental phenomena emergent from these patterns of brain activity are working models of self in relation to others—interpersonal schemas. As Safran (1990) has elegantly described, interpersonal schemas, a critical treatment target of cognitive behavioral therapies, may be more rigorously and fruitfully understood as elaborations of attachment characteristics defining these working models in terms of generalized scripts for interpersonal interaction. Such scripts describe a pattern of cognitive attributions or appraisals regarding relation of other to self *and* affective/behavioral responses adapted to preserve interpersonal relatedness based on that attributional frame. Such appraisals and affective/behavioral responses constitute central elements of what therapy might work on.

The general valence of the script, schema or working model may then be thought of as a level of attachment security (vs. fearfulness/disorganization). Where attachment is secure, there is a working expectation that appeals for relatedness will produce a positive and adequate response from the attachment figure. Such an expectation is based on a globally positive sense of self and other. Disner et al. (2011) suggest that in depression negative schema valence is a product of an imbalance of influence favoring subcortical/emotion processing regions over top-down cognitive control networks. Similarly, on a neural level, the association between attachment security and valence system function has been documented in a number of studies. Correlations between anterior cingulate gyrus (valence system) activity in response to attachment related stimuli has been correlated with attachment security in studies using narrative measures (AAI coherence of mind; Galynker et al., 2012), as well as with self-report attachment measures (DeWall et al., 2012), and indirect proxies such as romantic relationship length (Eisenberger et al., 2011). Anterior cingulate activity in response to attachment-relevant stimuli has likewise been correlated with level of (Zhang et al., 2011) and improvements in (Buchheim et al., 2012) depression.

The general tendency of the script, schema, or working model to ascribe low or high salience to interpersonal relatedness and accordingly to regulate intensity of interpersonal relatedness downward (deactivating) or upward (activating) may then be thought of as the ‘dismissiveness versus preoccupation’ attachment characteristic (Mikulincer et al., 2003). Previous studies (using the RSQ) have found attachment dismissiveness to correlate negatively with primary somatosensory and lateral prefrontal cortex (PFC) activity in response to sad faces (Suslow et al., 2009) and to associate with reduced processing of performance-incongruent social feedback among adolescents in medial and subcortical emotion processing structures (amygdala, caudate, anterior cingulate and insula; Vrticka et al., 2014). Similarly, listening to biographical narratives characteristic of dismissive attachment appeared to reduce subsequent functional connectivity in an empathy-associated network hub (Borchardt et al., 2015). Thus, at one end of the spectrum, persons with dismissive attachment have hypoactive attention and response to emotional input from others, while at the other end those with preoccupied attachment tend to over-respond.

### Measurement of Attachment

Assessments of adult attachment fall into two basic categories: observer-rated narrative measures of verbal behavior in response to a variety of stimuli, and self-report scales (Roisman et al., 2007). The Adult Attachment interview (AAI), belonging to the former category, is considered to be the gold standard of adult attachment assessment, but, being an extensive semi-structured interview requiring high-level training to administer and score, it is expensive, complex, and time consuming. The AAI assessment of attachment is based on verbal behavior, and examines attachment as manifested by the pragmatic structuring (Grice, 1975) of verbal behavior rather than by its overt content (George et al., 1996). As such, it is regarded as assessing implicit processes (Jacobvitz et al., 2002).

In contrast, the Relationship Scales Questionnaire (RSQ) is a popular self-report assessment of attachment which is quick and simple to score. However, it appears to measure different constructs from the AAI despite similarities in nomenclature. For example, Roisman et al. (2007) report that AAI security associates with the “Big Five” personality trait of *conscientiousness*, while RSQ security associates most strongly with *extroversion* and low levels of *neuroticism*. The RSQ assessment of attachment is based on the subjects’ overt, explicit representations and assessments of their behavior in relationships. Further, while the RSQ inquires about subjects’ experiences with relationships in general, the AAI focuses on subjects’ verbalizations of recollections of childhood experiences with their parents (George et al., 1996).

### Measurement of Attachment Dimensions

In this study we consider a dimensional approach, which characterizes attachment along two distinct dimensions—security-fearfulness and dismissiveness-preoccupation (Stein et al., 2002) that are equivalent to the anxiety (vs. security) and avoidance (vs. preoccupation) dimensions argued for by Kurdek (2002) for the RSQ.

The AAI conceptualization of security (vs. fearfulness/disorganization) is centered on the ability to maintain narrative organization and coherence in recounting memories of early attachment relationships (George et al., 1996) whereas the RSQ conceptualization of security (vs. fearfulness; Griffin and Bartholomew, 1994a) centers on self-report of trust in and comfort with attachment relationships (e.g., “I am comfortable depending on other people.” and “I am comfortable having other people depend on me.”; Kurdek, 2002). Both these approaches might be viewed as reflecting the global valence of the working model of self-other relations. The AAI approach, however, emphasizes narrative coherence as a marker of organization of the relational working model. Ethologically based attachment theory posits an over-all positive valence of primary self-other relation development through the ‘good enough mother’ who provides an adequately attuned and responsive holding environment for the developing infant as a baseline condition for development of a coherent and organized attachment representation (Winnicott, 1960). Thus, the AAI-approach to attachment security may be viewed as reflecting positive valence of self-other relational models implicitly. The RSQ approach, on the other hand, might be viewed as reflecting positive valence of self-other relational models as represented in explicit attributions.

The AAI conception of dismissiveness (vs. preoccupation) focuses on subjects’ verbal behavior, which deemphasizes relationships by providing inadequate, unconvincing, or even contradictory experiential evidence for qualitative assessments of those relationships (e.g., “My mother was wonderful; we did the usual things...I don’t remember anything in particular.”). At the other extreme, the AAI conception of preoccupation as manifest in verbal behavior focuses on over-emphasis of relationships in angry contexts through such features as over-inclusiveness, calling on the interviewer to join the speaker in the experience recounted, and re-living of these experiences. The RSQ conception of dismissiveness, on the other hand, focuses on low (vs. high) subjective desire for attachment and emotional proximity (e.g., “I am comfortable without close emotional relationships.”; Kurdek, 2002). Both these approaches might be viewed as reflecting diminished salience of self-other relations at the dismissive end of the spectrum. Again, the AAI might be understood as reflecting the behaviorally implicit salience, while the RSQ reflects the cognitively explicit salience of self-other relations.

While the face validity of the RSQ is quite strong, correlations between the RSQ (and other self-report scales of adult attachment) and the AAI (and other observer-rated measures of adult attachment) have been consistently low (Roisman et al., 2007). Nonetheless, both AAI and RSQ demonstrate predictive validity for behavioral observations predicted by attachment theory (Shaver and Mikulincer, 2004; Roisman et al., 2007; Ravitz et al., 2010). Thus, current evidence suggests that each assesses independent but clinically meaningful aspects of adult attachment.

### Study Aims

Understanding how these divergent but meaningful measures of attachment correlate with neural activity associated with processes pertinent to psychotherapy may thus shed light on the aspects of attachment they capture and explain what it means to find change in repeated measurement on these measures over the course of psychotherapy. In the present study, we examine the neural activity specific to conscious appraisal of subjects’ mothers (using the contrast between active appraisal and neutral viewing conditions) and aim to clarify how different approaches to measuring attachment might correlate with different elements of that brain activity.

Namely, insofar as the AAI (both in theory and overtly) relies on implicit markers of attachment representation, and thus is liable to assess primarily the implicit (affective/behavioral response) processes involved in the function of the internal attachment model, our first hypothesis is that this measure will correlate with brain activity associated with implicit/interoceptive brain function; we expect distribution of this activity to be preferentially associated with default mode network (DMN) and subcortical structures (Northoff et al., 2006; Northoff and Panksepp, 2008; Sajonz et al., 2010; Oosterwijk et al., 2015).

As the RSQ overtly captures conscious/explicit attributions, and thus is liable to assess primarily the explicit processes involved in the function of the internal attachment model, our second hypothesis is that RSQ measures will correlate with brain activity primarily in regions associated with conscious/expressive brain function and thus will preferentially identify executive frontal network (EFN) and generally cortical rather than subcortical structures (Northoff et al., 2006; Northoff and Panksepp, 2008; Sajonz et al., 2010; Oosterwijk et al., 2015).

To this end we examine the distributions of brain activity specific to conscious appraisal of a primary attachment figure correlating with the AAI and RSQ measures in relation to their basis in DMN vs. EFN networks as well as cortical vs. subcortical regional masks.

Further, we explore how brain activity specifically involved during explicit appraisal of a primary attachment figure varies with attachment security (vs. fearfulness/disorganization) and dismissiveness (vs. preoccupation), and how these dimensions interact with negative mood.

This study reanalyzes raw imaging data previously analyzed using other approaches in other publications. (Zhang et al., 2011; Galynker et al., 2012)

## Materials and Methods

### Participants

The study was approved by the Beth Israel Medical Center Institutional Review Board. All participants gave and signed a statement of informed consent. Basic study methodology has been previously described (Zhang et al., 2011; Galynker et al., 2012) and is reviewed below.

Physically healthy unmedicated depressed and non-depressed participants were recruited through online advertisements (craigslist.org), and screened by telephone and then in person by trained researchers. Participants were right-handed females aged 18–30 years who were able to understand and sign the informed consent and raised (birth to at least 14 years old) in a household with their biological mother. Potential subjects with current and lifetime substance abuse, history of head trauma or mental retardation, history of Schizophrenia, Schizoaffective Disorder, OCD, current suicidality or serious medical illness, or past year use of psychotropic medications were excluded.

### Instruments and Subject Evaluations

*The Mini-International Neuropsychiatric Interview (MINI)*, a short structured diagnostic interview for DSM-IV and ICD-10 psychiatric disorders, was used to establish subjects’ clinical diagnosis of depression and exclude other major psychiatric disorders (Sheehan et al., 1997, 1998). All MINI evaluations were conducted in the research office at the Beth Israel Medical Center 1–4 weeks prior to the scan.

#### Measures of Mood

*The Beck Depression Inventory II (BDI-II)* was used to assess depression (Beck et al., 1996a). Cronbach’s alpha for the BDI-II was 0.92 for outpatients and 0.93 for a non-clinical sample (Beck et al., 1996b).

*The Beck Anxiety Inventory (BAI)* was used to assess anxiety (Beck et al., 1988). Cronbach’s alpha for the BAI was 0.94 for outpatients (Fydrich et al., 1992) and 0.92 for a non-clinical sample (Osman, 1993).

As BDI and BAI scores in our sample were highly correlated (*r* = 0.85) a composite score of depression and anxiety (BDI + BAI) was used in subsequent analyses as a control for mood. At a cut score of 15, this composite measure had 100% sensitivity and 93% specificity for MINI diagnosis of depression in the study sample.

### Measures of Attachment

Attachment characteristics were assessed with the *Adult Attachment Interview (AAI)* and the *Relationship Scales Questionnaire-General (RSQ)*.

The *AAI* is a validated narrative assessment of attachment based on a structured semi-clinical interview focusing on early attachment experiences and their effects (George et al., 1996). AAI interviews were administered, videotaped, and transcribed by trained baccalaureate level research assistants. Research assistants were trained by an AAI-institute trained MEd researcher. Research assistants watched videotaped AAI interviews and performed practice interviews prior to interviewing study subjects. Interview transcripts were scored by AAI-training institute trained and certified PhD psychologist raters (Bakermans-Kranenburg and van Ijzendoorn, 1993).

From these interviews, and following the approach of Roisman et al. (2007) in their comparison of the AAI and RSQ, the Coherence of Mind index is derived as a measure of *attachment security*, with values ranging from 1 to 9. Scores (henceforth referred to as ‘AAI security scores’) 6–9 indicate secure attachment, scores 1–3 indicate insecure attachment and scores 4–5 are indeterminate (George et al., 1996). The coherence of mind index provides a conceptually unified core index of the implicit aspect of attachment security (George et al., 1996; Roisman et al., 2007).

A rating of *dismissiveness* ranging from -2 (most preoccupied) to +2 (most dismissing) was assigned based on attachment categorizations using the D/E/F/U/CC categorization system (George et al., 1996). Subjects’ categorical attachment type ratings and their derived ‘dismissiveness’ scale scores are shown in Supplementary Table S1. Subjects classified with an F subtype were scored between -1 and +1 on the Dismissiveness scale based on the description of the various F subtypes as either resembling the D (dismissing) type (+1), the E (enmeshed/preoccupied) type (-1), or the prototypical secure type (0). Subjects with a D-categorization were scored +2, and subjects with an E-categorization were scored -2. Subjects with a U (unresolved) or CC (cannot classify) categorization were scored according to the dominant D/E/F category accompanying the CC or U designation.

*The RSQ* is a well-validated 30-item, 5-point Likert scale, self-report questionnaire. From this scale, four subscale scores are derived – Secure, Fearful, Dismissing, and Preoccupied (Griffin and Bartholomew, 1994b; Kurdek, 2002). Using these subscale scores, composite dimensional Security and Dismissiveness scores were derived by subtracting Fearful from Secure score, and by subtracting Preoccupied from Dismissing score, respectively. This approach has yielded good reliability with Cronbach’s alphas of 0.85 and 0.81, respectively (Roisman et al., 2007).

Mood and attachment assessments were administered on the morning of the scan at the Hatch Imaging Center at Columbia Presbyterian Medical Center.

### Scanning Protocol

Stimuli were color photographs of the subject’s mother (M), a close female friend (F) and two strangers, one age matched to the mother and the other to the friend (S1 and S2). In the present study, however, data from friend and stranger stimuli was not used. The subject selected the photographs (straight on, shoulders up, taken within the past year) as most characteristic of the person being represented. Stranger photographs were selected from other subjects’ mother and friend photographs. Four different photographs of each person were provided. All images were processed using Photoshop to conform to approximately uniform head size, brightness, and contrast, and backgrounds were blacked-out.

There were four 12.6-min fMRI scans per subject. Each scan consisted of three blocks. For each block one of three tasks was defined for the subject with a written prompt. At the beginning of each block, this prompt was shown for 10 s. The prompts were: “How much can you relate to this picture?” (Relatedness task), “How pleasant do you feel when you look at this picture?” (Valence task), and “Press any button when you see the picture” (Passive/Neutral task). Each block consisted of 16 trials, with a picture viewed through goggles for 4 s. During this time subjects used their right hands to rate pictures according to the prompt by a recorded button-press. Ratings were on a 1–4 Likert (1–2 = negative to neutral, 3–4 = positive-very positive). Pictures were followed by a fixation-cross viewed passively by the subject for 10 s. Both type of picture and sequence of task were pseudo-randomized.

### fMRI Acquisition, Experimental Paradigm, and First Level Analysis

Scanning was performed on a Philips Intera 3T scanner using a Philips SENSE head coil (gradient echo EPI, TR/TE = 2 s/25 ms, 77° flip angle. Voxel size was 2 mm × 2 mm × 3 mm). Functional imaging data were preprocessed and analyzed with FSL (FMRIB Software Library; Smith et al., 2004). Motion correction parameters and global average of the BOLD for white matter were entered as covariates to control for movement and global BOLD signal fluctuation. Images were smoothed with a 9-mm FWHM Gaussian kernel.

Having found Mother stimulus to be the most pertinent in assessing attachment-related brain activity (Zhang et al., 2011; Galynker et al., 2012), in the present study, only data from viewing mother images was used. Thus, there were three relevant event related models: viewing images of mother (M) in each of three viewing conditions (Salience, Valence, and Neutral viewing). The models were convolved with the canonical hemodynamic response function. Two contrasts, Salience minus Neutral (henceforth referred to as Salience for concision), and Valence minus Neutral (henceforth referred to as Valence for concision) were applied and were averaged using fixed effects analysis. These served as the fMRI input for subsequent regression analyses. Significance of main-effects of contrast ROIs in whole brain analysis was assessed using FSL using a primary threshold *p* = 0.01, and a cluster-size probability threshold of *p* = 0.05, which provides cluster-extent based probability thresholding to correct for multiple comparisons.

### fMRI Second Level Analysis

We examined two regression models on the main effects of two contrasts for Mother images: Salience-Neutral task, and Valence-Neutral task, regressing each attachment measure against voxel contrast for the two contrasts above. Thus there were four regression models in total with two predictors of interest each, generating eight sets of ROIs:

(1a) AAI secure, covaried for AAI dismissing and BDI + BAI scores, (1b) AAI dismissing, covaried for AAI secure and BDI + BAI scores, (2a) RSQ secure, covaried for RSQ dismissing and BDI + BAI scores and (2b) RSQ dismissing, covaried for RSQ secure and BDI + BAI scores, regressed against Salience and Valence contrasts, respectively. Again, significance of ROIs in these whole brain analyses was assessed using FSL cluster-extent probability thresholding with a primary threshold *p* = 0.01, and a cluster-size probability threshold of *p* = 0.05.

### Analysis of ROI Distribution

To assess the distributions of AAI- and RSQ-correlated regions, the above ROIs (threshold voxel *z*-score > 1.96) derived from the second level analyses were intersected with Allen et al. (2011) anatomical masks for the internally oriented DMN, versus the more externally oriented, higher order processing, EFN (see Supplementary Figure S1). Additionally, the above ROIs were intersected with anatomical masks for cortical versus subcortical structures, generated using the Harvard-Oxford anatomical atlas (Desikan et al., 2006; see Supplementary Table S2). The proportion of AAI- and RSQ-correlated voxels falling within each pair of masks was then compared to the proportional distribution of available voxels between masks (i.e., DMN mask Voxel Count : EFN mask Voxel Count, and Cortical Mask Voxel Count : Subcortical Mask Voxel Count) using a χ^2^ test for each pair-wise comparison. For example, if two masks are of equal size, a null result for the AAI would find approximately equal numbers of voxels in the intersection of the AAI ROIs with each mask. On the other hand if the AAI measured properties more strongly associated with activity in the network represented by the first mask than the second, the intersection of AAI ROIs with the first mask would be significantly larger than with the second.

### Exploratory Analysis of Interaction Effects

For the exploratory analysis of interaction effects between mood and attachment measures, mood and attachment measures were median-centered and the relevant interaction term was added to each of the above models; for example, ‘Mood x AAI security’ was added to the model with Mood, AAI security, and AAI dismissiveness as covariates. Again, significance of ROIs in these whole brain analyses was assessed using FSL cluster-extent probability thresholding with a primary threshold *p* = 0.01, and a cluster-size probability threshold of *p* = 0.05.

## Results

### Sample Characteristics

The demographic and behavioral characteristics of the study sample have been described previously (Zhang et al., 2011; Galynker et al., 2012) and are summarized in **Table 1**.

**Table 1:** Behavioral and demographic characteristics of the sample.

Subject characteristics		
**Socio-demographic characteristics**	**Mean**	***SD***
Age (years)	24.5	2.9
Years of education	16.6	2.0
% of days in past year having contact with Mother^1^	49	39

Race/Ethnicity	***N***	**%**
Caucasian	21	75
African American	1	3.6
Hispanic/Latino	3	10.7
Asian	2	7.1
Other^2^	1	3.6

**Behavioral characteristics**	**Mean**	***SD***

Mother salience rating	3.6	0.6
Mother valence rating	3.4	0.8

*^1^% of the 365 days of the past year on which subject spoke with or saw Mother. ^2^Pacific Islander, Alaskan Native or Native American.*

### Measures of Attachment

For both the AAI and RSQ, attachment security and dismissiveness were independent of one another (correlations near zero), and both have moderate negative correlations with depression/anxiety. See **Table 2**.

**Table 2:** Pairwise Intercorrelations of AAI and RSQ attachment measures, and correlations with Depression/Anxiety.

Inter-Correlations of attachment measures					
	**AAI secure**	**AAI dismissing**	**RSQ secure-fearful**	**RSQ dismissing-preoccupied**	**Depression and Anxiety (BDI + BAI)**

AAI secure	1				-0.29
AAI dismissing	0.09	1			-0.36
RSQ secure-fearful	0.25	0.03	1		-0.47
RSQ dismissing-preoccupied	0.22	0.05	-0.05	1	-0.18

### Main Effects of Contrasts

**FIGURE 2:**
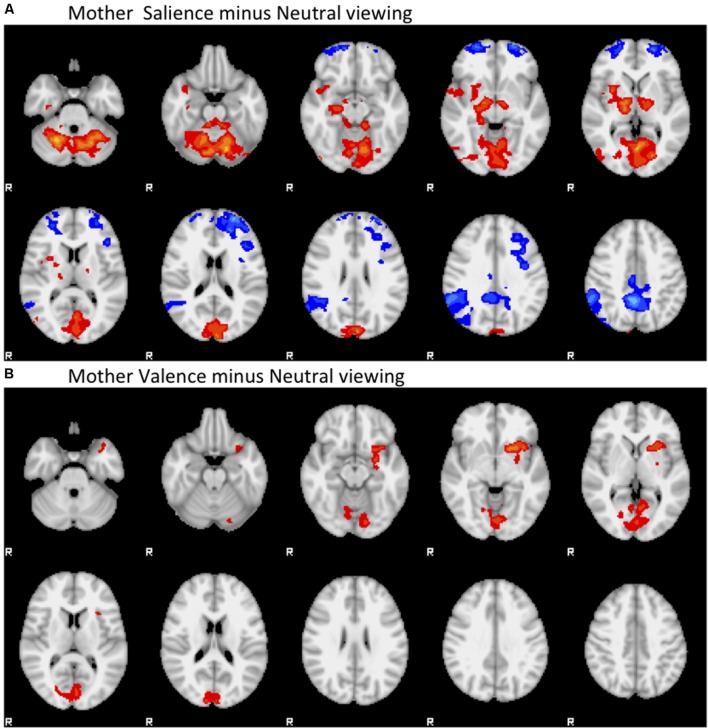
**Main Effects of contrast. (A)** Mother Salience minus Neutral viewing. **(B)** Mother Valence minus Neutral viewing. Negative contrasts are colored in the blue range, positive contrasts are colored in the red range. Contrasts start at *Z* = 1.96, colored dark blue or dark red, and range up to *Z* = 6, colored cyan or yellow.

*Active salience processing* associated with bilateral decreases in orbital PFC and posterior cingulate activity, and increases in thalamo-striatal activity and cerebellar and visual cortex (Brodmann Area 18) activity compared to passive viewing. *Active valence processing* also associated with bilateral increase in thalamo-striatal activity, posterior cingulate and visual cortex activity (Brodmann Areas 18, 19), cerebellar activity, and also notably associated with increased left insula activity compared to passive viewing, but was not associated with relative suppression of PFC activity. See **Figure 2**.

### DMN vs. EFN Activity Correlating Significantly with Attachment Measures

**FIGURE 3:**
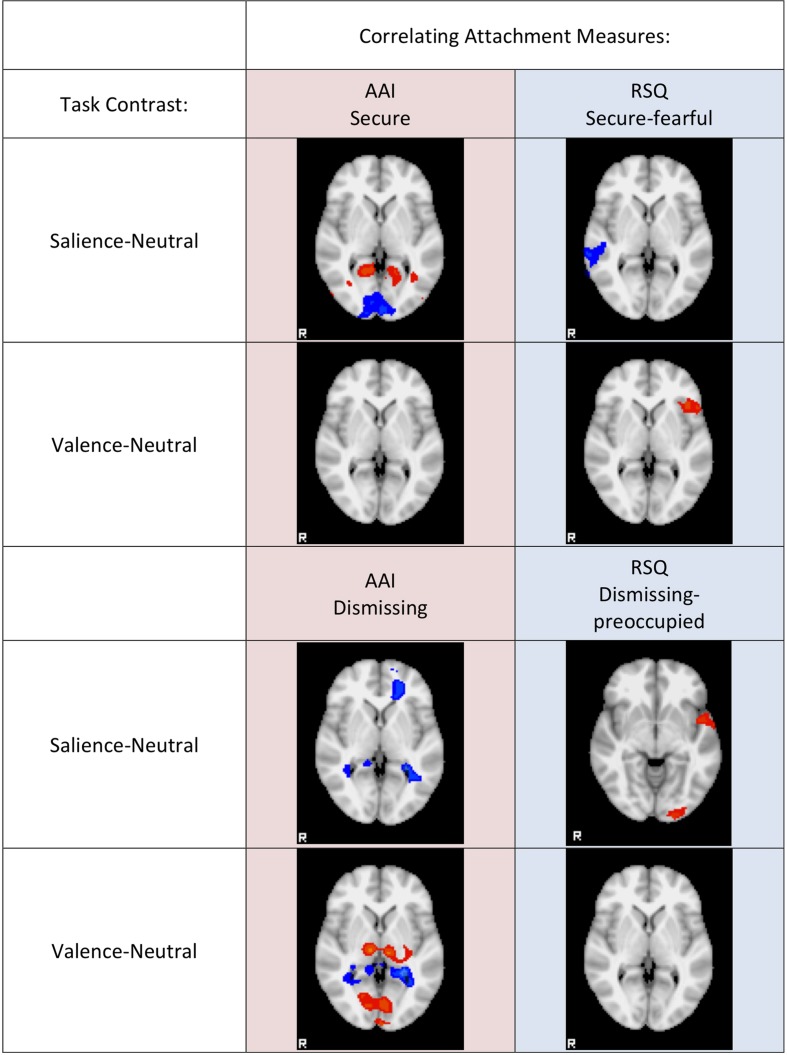
**Qualitative Visual summary.** Representative slices are shown for each Attachment measure (columns) regression against Mother viewing task contrast (rows). Negative correlations are colored in the blue range, positive correlations are colored in the red range. Correlation *z*-scores start at *Z* = 1.96 colored dark blue or dark red, and range up to *Z* = 6, colored cyan or yellow.

**FIGURE 4:**
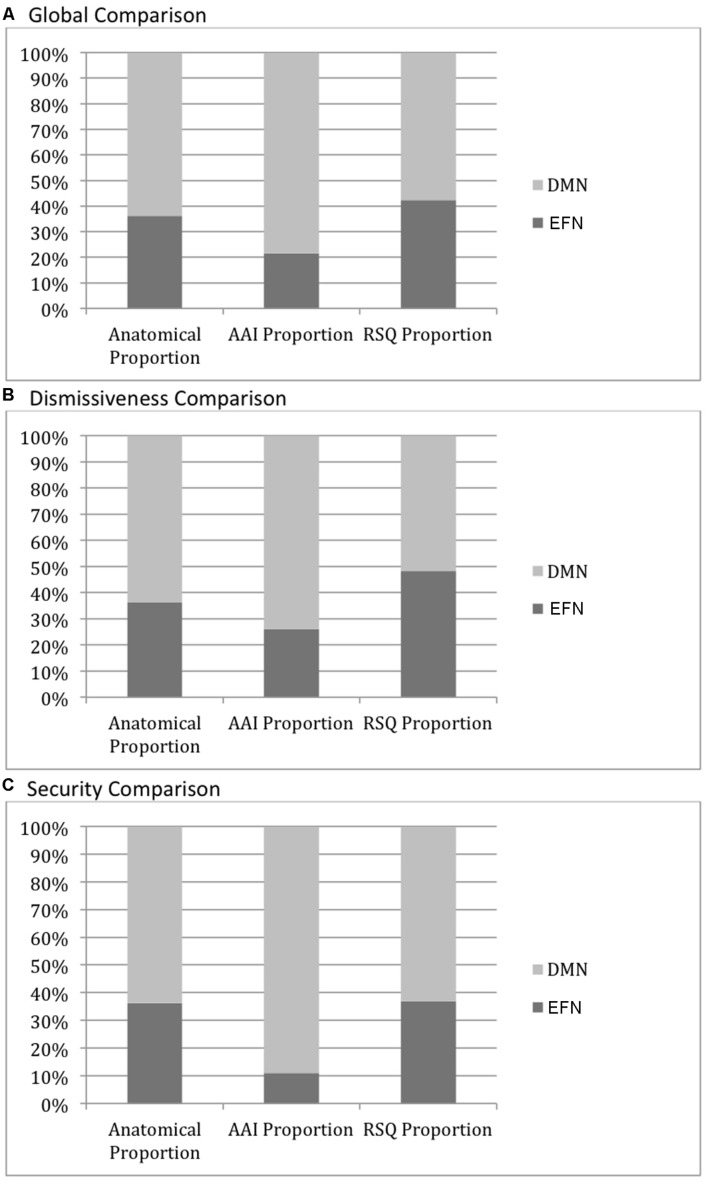
**Default mode network (DMN) vs. executive frontal network (EFN) cortical activity distributions.** The leftmost columns in each graph show the proportion of voxels in the DMN (light gray) vs. EFN (dark gray) masks, illustrating the relative size of each mask and the null-hypothesis distribution of voxels associated with each measure. The AAI and RSQ columns show the proportion of voxels significantly correlating with those measures falling within each mask. **(A)** DMN vs. EFN cortical activity: Global Comparison–all AAI/RSQ significant voxels. All pairwise proportion comparisons differ significantly at *p* < 0.0001. **(B)** DMN vs. EFN cortical activity: Dismissiveness Comparison–only AAI/RSQ Dismissiveness-measure significant voxels. All pairwise proportion comparisons differ significantly at *p* < 0.0001. **(C)** DMN vs. EFN cortical activity: Security Comparison–only AAI/RSQ Security-measure significant voxels. All pairwise proportion comparisons differ significantly at *p* < 0.0001 except RSQ vs. Anatomical (*p* > 0.05).

The DMN mask comprises 24287 voxels while the EFN network mask comprises 13841 voxels. Thus there is a 64:36 ratio of DMN to EFN voxels. Globally, looking at all activity significantly correlated with AAI measures (with threshold voxel *z*-score > 1.96 applied to the ROIs), there are 2898 voxels that fall within the DMN mask and 794 that fall within the EFN mask. Thus, in the AAI ROIs, DMN voxels are found at a 78:22 ratio to EFN voxels—significantly greater than the anatomical proportion [χ^2^(1) = 348, two-tailed *p* < 0.0001]. On the other hand, with respect to all activity significantly correlated with RSQ measures, there are 2396 voxels that fall within the DMN mask and 1763 that fall within the EFN mask. Thus, in the RSQ ROIs, DMN voxels are found at a 58:42 ratio to EFN voxels—significantly lower than the anatomical proportion [χ^2^(1) = 68, two-tailed *p* < 0.0001]. The results were similar, and significant at *p* < 0.0001, when AAI and RSQ Secure-Fearful and Dismissing-Preoccupied dimensions were compared separately, with the exception of RSQ security where the proportion was nearly identical to the anatomical proportion. See **Figure 3** for a qualitative visual summary, and see **Figure 4** for quantification of DMN vs. EFN distributions.

### Cortical vs. Subcortical Activity Correlating Significantly with Attachment Measures

Globally, looking at all activity significantly correlated with AAI measures vs. all activity significantly correlated with RSQ measures, the proportion of subcortically vs. cortically located activity was significantly higher than the anatomical proportion of subcortical vs. cortical voxels (15% subcortical vs. 85% cortical) for the AAI (31% subcortical vs. 69% cortical, χ^2^ 782.20, *p* < 0.0001), while the proportion was significantly lower than the anatomical proportion of subcortical vs. cortical voxels (3% subcortical vs. 97% cortical, χ^2^ 851.30, *p* < 0.0001) for the RSQ.

**FIGURE 5:**
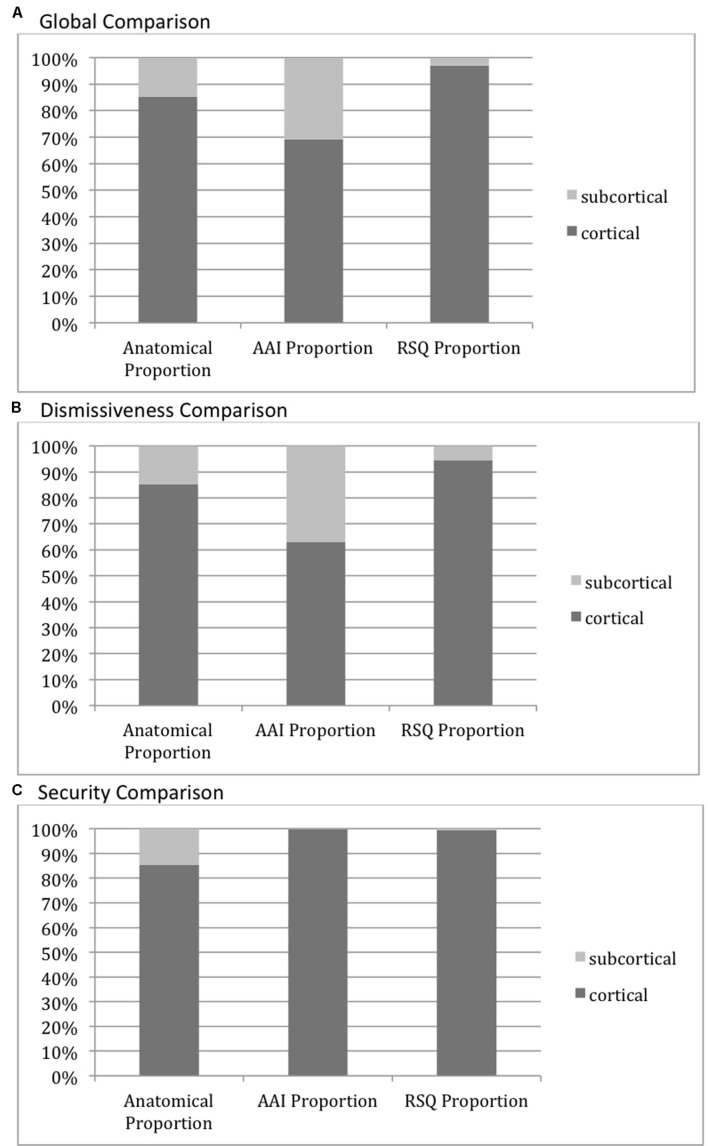
**Cortical vs. Subcortical activity distributions.** The leftmost columns in each graph show the proportion of voxels in the cortical (dark gray) versus subcortical (light gray) anatomical masks, illustrating the relative size of each mask and the null-hypothesis distribution of voxels associated with each measure. The AAI and RSQ columns show the proportion of voxels significantly correlating with those measures falling within each anatomical mask. **(A)** Cortical vs. Subcortical activity: Global Comparison–all AAI/RSQ significant voxels. All pairwise proportion comparisons differ significantly at *p* < 0.0001. **(B)** Cortical vs. Subcortical activity: Dismissiveness Comparison–only AAI/RSQ Dismissiveness-measure significant voxels. All pairwise proportion comparisons differ significantly at *p* < 0.0001. **(C)** Cortical vs. Subcortical activity: Security Comparison–only AAI/RSQ Security-measure significant voxels. Pairwise proportion comparisons with anatomical proportion differ significantly at *p* < 0.0001. AAI vs. RSQ proportions do not differ significantly.

However, this global finding was not repeated uniformly across attachment dimensions. Cortical versus subcortical distributions did not differ significantly between AAI and RSQ in the Secure-Fearful dimension, with both measures having overwhelmingly cortical activity associations (99.8 and 99.4% of significant voxels, respectively, *p* > 0.05 for comparison of AAI vs. RSQ proportions, *p* < 0.0001 for both comparisons with the anatomical proportion). In the Dismissing-Preoccupied dimension, however, the global results were repeated with 37% of AAI vs. 5% of RSQ correlated activity within the subcortical mask (two-tailed *p* < 0.0001 for both comparisons with the anatomical proportion). See **Figure 5**.

### Specific Brain Structure Activities Correlating with AAI and RSQ Measures of Attachment

Coordinates of all peak voxels significant at the *p* < 0.005 level in clusters with extent based significance *p* < 0.05 are listed in Supplementary Tables S3–S5.

AAI security was significantly correlated with activity in the right parahippocampal gyrus—a brain region associated with social cognition including empathy and the interpretation of non-verbal communication (Vollm et al., 2006; Rankin et al., 2009), as well as the right posterior cingulate and fusiform gyri—brain regions associated with empathy (Vollm et al., 2006), and facial recognition (Haxby et al., 2000), respectively. It was anti-correlated with activity in the cuneus bilaterally—an occipital brain region found to be activated by explicit attention to negative affect (Sander et al., 2005).

Relationship Scales Questionnaire security on the other hand was significantly anti-correlated with right middle temporal gyrus and left lateral PFC activity—brain regions associated with semantic processing of visual emotional cues, and explicit affect regulation, respectively (Ochsner and Gross, 2005; Ochsner et al., 2012).

Adult Attachment Interview dismissiveness was significantly correlated with activity in the right cuneus and left lingual gyrus—an area associated with facial recognition, as well as the thalamus bilaterally—a subcortical region involved in affective processing, including maternal response to infant distress (Noriuchi et al., 2008; Diener et al., 2012). AAI dismissiveness also demonstrated significant negative correlations with activity in the corpus callosum—the major inter-hemispheric tract of white matter fibers, left medial frontal gyrus, and right anterior cingulate—a region involved in negative affect regulation (Etkin et al., 2011), as well as the parahippocampal gyri, and bilateral temporal lobe white matter tracts.

Relationship Scales Questionnaire dismissiveness, demonstrated significant negative correlations with cerebellar activity, left superior temporal gyrus, right anterior cingulate and right cuneus activity.

### Exploratory Comparison of Interaction Effects between Mood and AAI vs. RSQ Measures of Attachment

Interaction effects with mood were found for both the AAI and RSQ, primarily in brain regions involved in regulation of affect as well as semantic processing and memory. AAI measures of attachment characteristics demonstrated interactions with mood in brain regions involved in regulation of affect as well as visual attention to affective stimuli and memory retrieval. AAI security demonstrated enhancing interactions with negative mood in the caudate tail—a region involved in guiding visual attention (Yamamoto et al., 2012), and in the right temporal lobe. A negative interaction was found in the right cuneus—where activity is associated with attention to negative affect (Sander et al., 2005)—suggesting that heightened activity in this region associated with dysphoric mood is attenuated by more secure attachment. A negative interaction was found in the right superior frontal gyrus as well—where activity is associated with conscious regulation of affect (Beauregard et al., 2001)—suggesting, perhaps, that securely attached subjects were less able to regulate affect during maternal appraisal when depressed. Finally, AAI security demonstrated a negative interaction with mood in the right middle temporal gyrus, a region associated with semantic processing of emotional visual stimuli (Ochsner and Gross, 2005).

Negative interaction effects were also found between mood and AAI dismissiveness in the right medial frontal gyrus, and bilateral medial somatosensory association cortices including the precuneus; these areas have been associated with affect regulation, and self-related mental imagery and episodic memory retrieval, respectively (Cavanna and Trimble, 2006; Fransson and Marrelec, 2008).

For RSQ measures of attachment styles, interaction effects were also found in brain regions associated with semantic processing, memory, and affect regulation. RSQ security demonstrated positive interactions with mood in Wernicke’s area. As with AAI measures, RSQ security demonstrated negative interactions with mood in the right precuneus and middle temporal gyri. RSQ dismissiveness also demonstrated negative interactions with mood in the precuneus, as well as neighboring somatosensory association cortex areas. Surprisingly, RSQ dismissiveness also demonstrated an enhancing interaction with dysphoric mood in the supplementary motor area—a brain region that has been implicated in empathy (Borchardt et al., 2015).

Significant clusters are illustrated in Supplementary Figure S2. Coordinates of all peak voxels significant at the *p* < 0.005 level in clusters with extent based significance *p* < 0.05 are listed in Supplementary Table S6.

## Discussion

We commonly ask patients how they felt toward or were made to feel by others, especially significant others, with whom attachment plays an important relationship-shaping role, such as parents, romantic partners, children, and ourselves (as their therapists; Schore, 2003). Thus, the conscious appraisal of attachment figures’ affective significance is a task with substantial ecological validity for a wide variety of psychotherapies, as making emotional appraisals conscious and explicit is a highly prevalent process in many. The process of such conscious affective appraisal is liable both to be influenced by attachment configuration and to be a therapeutic factor in the psychotherapy process (Schore, 2003).

This study is the first to compare self-report (explicit) and observer-rated narrative (implicit) measures of adult attachment in terms of brain activity. We compared the distributions of brain regions where activity associated with appraisal of a primary attachment figure correlated with either the AAI—an observer rated narrative measure, relying on non-conscious manifestation of attachment representations in verbal behavior, or, the RSQ—a self-report measure of adult attachment relying on conscious appraisal of attachment styles. Further, we examined the correlations between neural activity associated with explicit attachment-figure appraisal and attachment-dimensions as measured by the AAI and RSQ, as well as their interactions with negative mood.

Our results are pertinent to psychotherapy research in a number of ways. First, we obtain findings supporting the supposition that implicit and explicit assessments of attachment do in fact correspond to assessment of implicit and explicit components of the complex mental functions characterizing attachment configurations, with AAI assessment of attachment representation correlating with activity preferentially distributed in brain regions (the DMN and subcortical structures) associated with interoceptive and core affective experience, and RSQ assessed attachment style measures correlating with activity preferentially distributed in brain regions associated with exteroceptive and higher-order explicit processing of such affective information (EFN networks and cortical structures; Northoff et al., 2006; Sajonz et al., 2010). Second, we identify neural substrates whose activity during explicit appraisal of attachment-related affect is sensitive to variation with specific measures and dimensions of attachment characteristics. Finally, we demonstrate regions where negative mood and attachment characteristics interact in their effects on brain activity during attachment-figure appraisal.

In accordance with our first hypothesis, brain activity correlating with AAI measures of attachment was found disproportionately in DMN and subcortical brain regions, as would be expected for the measure assessing the pre-conscious/interoceptive aspects of attachment. In accordance with our second hypothesis, brain activity correlating with RSQ measures of attachment was found preferentially in Attentional/Frontal control areas with very little involvement of subcortical structures, as might be expected for this instrument measuring conscious/explicit aspects of attachment.

Furthermore, these findings generally held across orthogonal dimensions of attachment—the Secure-Fearful/Disorganized and Dismissing-Preoccupied dimensions—indicating that these findings are independent of rotation of attachment dimensions.

We found that RSQ-assessed attachment security was significantly anti-correlated with activity in the right middle temporal gyrus (involved in semantic processing of visual emotional cues) and in the left lateral PFC (involved in explicit affect regulation) during deliberate appraisal of subjects’ mothers (Ochsner and Gross, 2005; Ochsner et al., 2012). This reduction in activity among more RSQ-securely attached subjects may indicate less effortful explicit processing of relational information. This is consistent with the teleology of secure attachment as resulting in a working model of the attachment figure as reliable and safe and thus requiring less explicit self-regulation of affect to be (mentally) approached. AAI-assessed security on the other hand was associated with enhanced activity in midline regions involved in empathy suggesting more active implicit processing of relational information. This finding is consistent with the etiology of secure attachment as the product of an empathic relationship between parent and offspring.

Interactions with severity of dysphoric mood were found for both the AAI and RSQ, primarily in brain regions involved in regulation of affect as well as semantic processing and memory. Negative interactions between attachment security and dysphoric mood were found for both AAI and RSQ measures in the middle temporal gyrus—a region associated with facial memory (Brent et al., 2016) and facial affect processing (Pohl et al., 2013). Notably this region is also involved in preferential parental responses to their own over others’ infants (Atzil et al., 2012). Negative interaction effects on activity in this region suggest that reduction of activity here associated with depression (Vizueta et al., 2012) may be heightened by less secure and more preoccupied attachment characteristics.

Notably, also, both AAI and RSQ measures of dismissiveness exhibited negative interactions with mood in the precuneus. This brain region has been found to be an important hub of the DMN where it may serve to integrate self-referential thought and episodic memory retrieval (Fransson and Marrelec, 2008). Such a function seems to be highly pertinent to the conscious appraisal of the affective impact of attachment figure images. This negative interaction effect may be due to opposing cognitive biases associated with dismissing attachment and depression. Dismissing vs. preoccupied attachment is characterized by inhibited vs. enhanced recall of negative valence interactions on the one hand, and derogation of the value (positive valence) of connection on the other. Meanwhile depression is characterized by an attentional bias for negative information. Thus, perhaps, increased recall of negative interactions with attachment figures by depressed subjects as they attempt to evaluate the affective impact of attachment figure images is attenuated by more dismissing attachment and amplified by more preoccupied attachment.

These findings have both theoretical and practical implications. On a theoretical level, our results provide evidence that the overt contents of AAI and RSQ assessments match the actual constructs assessed by each. That is, the implicit assessment (AAI) overtly assesses non-conscious/implicit processes which are products of a “core self,” while the self-report scale (RSQ) assesses (necessarily) conscious/explicit appraisals of emotion and behavior, and thus produces an assessment of “higher-order” cognitive aspects of relational function (Northoff et al., 2006; Sajonz et al., 2010). Moreover, these “higher-order” aspects of adult attachment are likely to be developmentally secondary to the AAI-assessed ones. Because of the iterative pattern of brain development, with globally burgeoning development in infancy, and continuing development through late adolescence (Shaw et al., 2008; Gilmore et al., 2012; Alcauter et al., 2014), the trajectory of development of core vs. higher-order self-functions is best examined through cognitive/behavioral rather than anatomical measures. In terms of cognitive/behavioral measures, a developmental hierarchy, with the emergence of, implicit core cognitive processes followed by explicit higher-order cognitive processes in childhood cognitive/behavioral maturation has been documented (Case, 1991; Howe et al., 1994; Howe and Courage, 1997). Thus, the attachment phenomena assessed by the RSQ may be viewed as the developmentally secondary and more externally oriented outcomes of the more primary internally oriented phenomena assessed by the AAI. Such a view would predict both the observed clinical relevance of both measures, and the limited correlations observed between them, as common primary processes are liable to find divergent secondary cognitive and behavioral expressions (Allen et al., 1998).

Considering attachment security as a dimension of relational script valence, one might relate this dimension to brain activity as a product of the intrinsic responsivities of positive vs. negative valence systems and compensatory activity of the systems that regulate them, as suggested, for example, by Disner et al. (2011) in relation to depression. In light of our findings, the AAI might be understood as more strongly related to those intrinsic responsivities while the RSQ might be understood as more strongly related to the activity of the regulatory system. Both systems, however, might be psychotherapeutic targets as well as moderators of intervention efficacy.

Considering attachment dismissiveness (avoidance) as a dimension of relational script salience, one might relate this dimension to brain activity as a product of the intrinsic responsivities of positive *and* negative valence systems and systems regulating attention to positive and negative social cues. Again, our findings suggest that the AAI measure might be considered as more strongly related to primary affective responsivity, while the RSQ might be considered as more strongly related to activity of the systems responsible for regulating attention to social information.

Our findings may thus also have important practical implications for psychotherapy study design and interpretation. For example, attachment characteristics are developed early in life and are largely conserved (Waters et al., 2000); this makes attachment a strong target for investigation as moderator of change processes and treatment outcomes in psychotherapy.

Several studies have examined moderation of psychotherapeutic outcomes by attachment characteristics, e.g., (McBride et al., 2006; Bagby et al., 2008). McBride et al. (2006) using the RSQ, found that with increasingly avoidant attachment, cognitive-behavioral therapy demonstrated increasing advantage over interpersonal psychotherapy in the treatment of major depression. Our findings bear on the interpretation of these results, given the author’s use of the RSQ. The observed interaction between attachment and treatment modality might be predicted on two distinct (though not mutually exclusive) theoretical grounds, as the authors note in their discussion. Namely, interpersonal therapy, by focusing on close relationships, might be too emotionally threatening to persons with high attachment avoidance, and thus elicit excess distress, or it may be cognitively dissonant for persons who explicitly devalue close relationships and result in poor treatment alliance (which has been robustly demonstrated to be an important predictor of treatment outcome; Horvath and Symonds, 1991). Our findings would support the latter interpretation based on the authors’ use of the RSQ and its primarily lateral cortical activity associations. Replication of McBride et al.’s (2006) findings using the AAI, which we found to associate significantly with medial and subcortical brain activity and thus be more liable to reflect core generation and regulation of emotion, might more strongly support the ‘excess emotional threat’ interpretation of their results.

Increases in attachment security can be important outcomes in psychotherapy (Travis et al., 2001; Levy et al., 2006). Moreover, such changes may be particular to the type of psychotherapy (Levy et al., 2006). Thus, in assessing the relative efficacy of specific therapeutic modalities in effecting attachment changes, the mechanism of action at the neuroactivity level should be related to the measure of outcome at the neuroactivity level. For example, transference focused therapy (TFP) focuses heavily on the development of reflective function (increasing interoception) as its mechanism of action (Clarkin et al., 2007). In turn, reflective function relies primarily on midline cortical structures (Buckner and Carroll, 2007). Thus our findings would indicate use of the AAI rather than the RSQ to assess attachment changes (Levy et al., 2006) in TFP. In contrast, schema focused therapy places significant emphasis on cognitive restructuring of patient schemata (Young et al., 2003; Kellogg and Young, 2006). Such deliberate cognitive restructuring may rely more prominently on processes such as deliberate reappraisals of negative emotions that bring in exteroceptively derived ‘factual data’ (e.g., ‘Mary always smiles when she sees me’). Such deliberate reappraisals rely heavily on lateral prefrontal cortical activity and activity in the area of the temporoparietal junction (Ochsner et al., 2002; Buhle et al., 2014). One might therefore predict that RSQ assessment of attachment change would be more sensitive to effects of such a treatment than an implicit/projective measure such as the AAI. Furthermore, these lateral cortical regions (subserving exteroceptive, RSQ-assessed relational functions) demonstrate significant and evolutionarily conserved connectivity (Mars et al., 2013) to the medial PFC (subserving interoceptive, AAI-assessed relational functions). Thus, one might expect RSQ-assessed changes in attachment to mediate AAI-assessed changes following cognitive therapies, and the reverse to be observed following transference or emotion focused therapies.

On the other hand, both therapies might be characterized by a common factor of repeated exposure to interactions that would be perceived attachment threats (e.g., presaging rejection or abandonment) in the context of an insecure script. By a process of reversal learning, given a strong working alliance where the feared rejection or abandonment is seen not to occur, such repeated exposure might decouple the interaction from the threat response. This learning might be driven in different stages by changes in explicit regulatory strategy and by attenuation of core affective responsiveness (or both processes may occur in simultaneous and complementary fashion). To understand how such changes occur over time, tasks and biological measurement that probe both regulatory functions and core affective responses should be used along with attachment measures such as the RSQ and the AAI, which might reflect, respectively, the contributions of each of these domains.

### Limitations

This study has a number of important limitations that should be considered in interpreting the findings. First, we examine only one narrative and one self-report measure of attachment. A detailed discussion of the range of adult attachment measures is beyond the scope of this paper, and so, the reader should note that there are several excellent reviews of adult attachment measures, Ravitz et al. (2010) being the most recent, to our knowledge. It is nonetheless worth considering here some other important exemplars of narrative and self-report measures of adult attachment. Among *narrative* measures of attachment, the Adult Attachment Projective (AAP; George and West, 2001) is the most prominent alternative to the AAI, and relies on analysis of narrative interpretations of a standardized set of attachment-related images (Ravitz et al., 2010). This feature has made it particularly valuable in the study of brain processes involved in adult attachment as AAP stimuli are easily presented during fMRI scanning. (See, for example, several of the articles in this special issue of Frontiers as well as (Buchheim et al., 2006, 2008). It is worth noting, however, that there is very strong convergence between AAI and AAP assessments (Ravitz et al., 2010), so our results for the AAI might be expected to apply to the AAP as well. Among *self-report* assessments of adult attachment, a recent meta-analysis comparing five commonly used dimensional measures, not including the RSQ, found the Revised Experiences in Close Relationships scale, which focuses on attachment in partnered (i.e., romantic) relationships, to have superior reliability with a stable two-factor structure (anxiety and avoidance) akin to our security and dismissiveness dimensions for the RSQ (Graham and Unterschute, 2015). Given modest correlations among self-report measures, it is possible that our results for the RSQ might not apply to other measures.

In addition to the limitation of using only one self-report and one narrative attachment measure, we also report on only one of several possible dimensional approaches to characterization of these assessments. Others have proposed other methods for assessment of attachment dimensions in the RSQ and AAI. However, our results document similar DMN versus EFN activity distributions across orthogonal dimensions within a measure but different distributions between measures. This suggests that the limitation involved in our choice of attachment dimension extraction from the measures used is more pertinent to the consideration of specific individual structures’ functional relations to a given measure than to the global comparison of the AAI versus the RSQ’s patterns of association with brain activity. In addition, it should be noted that dimensional approaches to interaction effects are sensitive to transpositions of the dimensional measures; to mitigate this issue we median centered our measures for interaction effect analyses so that zero would correspond approximately to the cut point between categorical notions of symptomatic and asymptomatic. However, in samples with different median scores, different results might be obtained.

A second limitation of the current study is the lack of assessment and control for the extent to which subjects’ mothers were their primary attachment figures. However, the requirement that subjects have been raised in a household with their mothers from birth until at least age thirteen significantly mitigates this limitation.

A third limitation is the absence of systematic activation of the attachment system by any threatening or distressing stimulus in the scanning paradigm, as such stimuli have been found to increase the salience of attachment figures (Mikulincer et al., 2002). While the cold and isolated conditions of an fMRI scan might serve this purpose, some studies suggest more attachment-specific stressors may be needed (Nolte et al., 2013).

Finally generalizability is limited both by the small sample size and the restriction to women aged 18–30 years.

## Conclusion

The AAI and RSQ measure different aspects of attachment with highly divergent associated brain activity. The AAI taps DMN and subcortical structure activity more extensively, while the RSQ taps EFN activity more extensively. Thus, the AAI may assess non-conscious ‘core-self’ and interceptive processes in attachment figure appraisal, while the RSQ captures higher-order cognitive aspects that integrate externally derived information. Common effects of AAI- and RSQ-measured attachment security on the impact of mood on deliberate affective appraisal of an attachment figure are consistent with the notion that the ‘core-self’-related and higher-order cognition-related processes tapped by each measure do indeed belong to a common attachment network.

## Author Contributions

All authors listed, have made substantial, direct and intellectual contribution to the work, and approved it for publication.

## Conflict of Interest Statement

The authors declare that the research was conducted in the absence of any commercial or financial relationships that could be construed as a potential conflict of interest.
