# Supplementary material for: Comparison of Brain Activity Correlating with Self-Report versus Narrative Attachment Measures during Conscious Appraisal of an Attachment Figure
Source: Front Hum Neurosci. 2016 Mar 14;10:90. doi: 10.3389/fnhum.2016.00090 (PMC4789543; doi:10.3389/fnhum.2016.00090)
Supplement: Supplementary file 1 [file Supplementary_Tables_1-6.DOCX]

**Supplementary tables:**

### Supp. Table 1.

| **AAI categorical classification and assigned Dismissingness scores for each subject** | |
| --- | --- |
| ***AAI attachment style category*** | ***Dismissingness*** |
| CC/F3/E2/E3 | -2 |
| E1 | -2 |
| E2 | -2 |
| E2 | -2 |
| E2/F5 | -2 |
| Ud(tr)/E2 | -2 |
| F4 | -1 |
| F4 | -1 |
| F4 | -1 |
| F4a | -1 |
| F4a | -1 |
| Ud(tr)/F4 | -1 |
| F3 | 0 |
| F3 | 0 |
| F3 | 0 |
| F3 | 0 |
| Ud/F3 | 0 |
| F1b | 1 |
| F2 | 1 |
| F2 | 1 |
| F2 | 1 |
| F2 | 1 |
| F3/F2 | 1 |
| Ud(tr)/F2 | 1 |
| Ds1 | 2 |
| Ds1 | 2 |
| Ds1 | 2 |
| Ds3 | 2 |

### Supp. Table 2. Anatomical Masks

| 1. **Subcortical Anatomical Mask Regions *** |
| --- |
| Label index=3 <x= +/-50 y=52 z=39> Thalamus |
| Label index=4 <x= +/-51 y=70 z=40> Caudate |
| Label index=5 <x= +/-56 y=67 z=34> Putamen |
| Label index=6 <x= +/-54 y=62 z=35> Pallidum |
| Label index=7 <x= +/-44 y=50 z=20>Brain-Stem |
| Label index=8 <x= +/-59 y=55 z=26> Hippocampus |
| Label index=9 <x= +/-57 y=62 z=25> Amygdala |
| Label index=10 <x= +/-50 y=69 z=32> Accumbens |
| * Cortical anatomical mask regions represent the union of the Harvard-Oxford atlas cortical regions |

Supp. Table 3: Main Effect of Contrast

**3a. Mother Salience>Neutral**

| Name | X | Y | Z | Z Score | P value | Cluster ID | Cluster Size |
| --- | --- | --- | --- | --- | --- | --- | --- |
| Right Cerebellum.Anterior Lobe.Gray Matter | 22 | -58 | -30 | 4.95 | 0.0000 | 1 | 11152 |
| Left Cerebrum.Occipital Lobe | -2 | -66 | 4 | 4.88 | 0.0000 | 1 | 11152 |
| Left Cerebellum.Anterior Lobe.Gray Matter | -22 | -62 | -30 | 4.46 | 0.0000 | 1 | 11152 |
| Left Cerebellum.Anterior Lobe.Culmen.Gray Matter | -16 | -58 | -20 | 4.43 | 0.0000 | 1 | 11152 |
| Left Cerebellum.Posterior Lobe.Declive.Gray Matter | -14 | -72 | -22 | 4.26 | 0.0000 | 1 | 11152 |
| Left Cerebrum.Occipital Lobe.Cuneus.Gray Matter.BA 18 | -2 | -90 | 22 | 4.03 | 0.0000 | 1 | 11152 |

**3b. Mother Salience<Neutral**

| Name | X | Y | Z | Z Score | P value | Cluster ID | Cluster Size |
| --- | --- | --- | --- | --- | --- | --- | --- |
| Left Cerebrum.Frontal Lobe.Superior Frontal Gyrus.Gray Matter.BA 9 | -32 | 54 | 24 | 4.45 | 0.0000 | 3 | 4214 |
| Left Cerebrum.Frontal Lobe.Superior Frontal Gyrus.Gray Matter.BA 10 | -24 | 56 | 20 | 4.43 | 0.0000 | 3 | 4214 |
| Left Cerebrum.Frontal Lobe.Superior Frontal Gyrus.White Matter | -20 | 60 | 2 | 3.83 | 0.0001 | 3 | 4214 |
| Left Cerebrum.Frontal Lobe.Middle Frontal Gyrus.White Matter | -24 | 58 | 0 | 3.77 | 0.0001 | 3 | 4214 |
| Right Cerebrum.Frontal Lobe.Medial Frontal Gyrus.White Matter | 22 | 62 | -4 | 3.74 | 0.0001 | 3 | 4214 |
| Right Cerebrum.Frontal Lobe.Superior Frontal Gyrus.White Matter | 26 | 64 | -6 | 3.70 | 0.0001 | 3 | 4214 |
| Right Cerebrum.Parietal Lobe.Supramarginal Gyrus.White Matter | 62 | -50 | 38 | 4.73 | 0.0000 | 2 | 2032 |
| Right Cerebrum.Parietal Lobe.Supramarginal Gyrus.White Matter | 46 | -46 | 32 | 3.78 | 0.0001 | 2 | 2032 |
| Right Cerebrum.Temporal Lobe.Superior Temporal Gyrus.White Matter | 62 | -56 | 24 | 3.27 | 0.0005 | 2 | 2032 |
| Right Cerebrum.Temporal Lobe.Superior Temporal Gyrus.Gray Matter.BA 39 | 60 | -62 | 30 | 3.19 | 0.0007 | 2 | 2032 |
| Right Cerebrum.Temporal Lobe.Angular Gyrus.Gray Matter.BA 39 | 44 | -76 | 36 | 2.87 | 0.0021 | 2 | 2032 |
| Right Cerebrum.Parietal Lobe.Cingulate Gyrus | 2 | -48 | 42 | 4.76 | 0.0000 | 1 | 1245 |
| Left Cerebrum.Parietal Lobe.Precuneus.White Matter | -10 | -58 | 42 | 3.30 | 0.0005 | 1 | 1245 |
| Right Cerebrum.Limbic Lobe.Cingulate Gyrus | 6 | -22 | 42 | 3.28 | 0.0005 | 1 | 1245 |
| Left Cerebrum.Parietal Lobe.Precuneus.White Matter | -14 | -48 | 38 | 3.05 | 0.0011 | 1 | 1245 |
| Left Cerebrum.Limbic Lobe.Cingulate Gyrus.White Matter | -14 | -32 | 42 | 2.64 | 0.0041 | 1 | 1245 |

**3c. Mother Valence>Neutral**

| Name | X | Y | Z | Z Score | P value | Cluster ID | Cluster Size |
| --- | --- | --- | --- | --- | --- | --- | --- |
| Left Cerebrum.Occipital Lobe.Lingual Gyrus | 2 | -78 | -4 | 3.63 | 0.0001 | 1 | 1929 |
| Left Cerebrum.Occipital Lobe.Lingual Gyrus.Gray Matter.BA 19 | -8 | -62 | 2 | 3.21 | 0.0007 | 1 | 1929 |
| Left Cerebellum.Anterior Lobe.Culmen of Vermis.Gray Matter | -4 | -64 | 0 | 3.08 | 0.0010 | 1 | 1929 |
| Left Cerebellum.Posterior Lobe.Declive.Gray Matter | -14 | -82 | -14 | 3.08 | 0.0010 | 1 | 1929 |
| Left Cerebellum.Posterior Lobe.Declive.Gray Matter | -16 | -82 | -18 | 3.02 | 0.0013 | 1 | 1929 |
| Right Cerebrum.Occipital Lobe.Cuneus.Gray Matter.BA 18 | 12 | -92 | 18 | 3.00 | 0.0013 | 1 | 1929 |

### Supp. Table 4: AAI vs RSQ measured Attachment Security Brain Activity

**4a. AAI security positive correlation with Salience contrast**

| Name | X | Y | Z | Z Score | P value | Cluster ID | Cluster Size |
| --- | --- | --- | --- | --- | --- | --- | --- |
| Right Cerebrum.Limbic Lobe.Parahippocampal Gyrus.Gray Matter.BA 19 | 20 | -46 | -4 | 4.11 | 0.0000 | 2 | 2221 |
| Right Cerebrum.Temporal Lobe.Middle Temporal Gyrus.White Matter | 38 | -74 | 26 | 3.73 | 0.0001 | 2 | 2221 |
| Right Cerebrum.Limbic Lobe.Posterior Cingulate.White Matter | 28 | -64 | 20 | 3.67 | 0.0001 | 2 | 2221 |
| Right Cerebellum.Anterior Lobe.Culmen.Gray Matter | 8 | -56 | -6 | 3.55 | 0.0002 | 2 | 2221 |
| Right Cerebrum.Occipital Lobe.Fusiform Gyrus.Gray Matter.BA 37 | 34 | -50 | -10 | 3.46 | 0.0003 | 2 | 2221 |
| Right Cerebrum.Occipital Lobe.Lingual Gyrus | 16 | -52 | 4 | 3.41 | 0.0003 | 2 | 2221 |
| Left Cerebellum.Anterior Lobe.Culmen.Gray Matter | -36 | -40 | -24 | 3.81 | 0.0001 | 1 | 1911 |
| Left Cerebrum.Occipital Lobe.Middle Occipital Gyrus.White Matter | -50 | -82 | 12 | 3.79 | 0.0001 | 1 | 1911 |
| Left Cerebrum.Temporal Lobe.Sub-Gyral.White Matter | -36 | -56 | 0 | 3.17 | 0.0008 | 1 | 1911 |
| Left Cerebrum.Occipital Lobe.Middle Occipital Gyrus.White Matter | -36 | -70 | 20 | 3.04 | 0.0012 | 1 | 1911 |
| Left Cerebrum.Temporal Lobe.Sub-Gyral.White Matter | -26 | -70 | 22 | 2.95 | 0.0016 | 1 | 1911 |

**4b. AAI security negative correlation with Salience contrast**

| Name | X | Y | Z | Z Score | P value | Cluster ID | Cluster Size |
| --- | --- | --- | --- | --- | --- | --- | --- |
| Right Cerebrum.Occipital Lobe.Cuneus.White Matter | 14 | -92 | 14 | 3.92 | 0.0000 | 1 | 1949 |
| Left Cerebrum.Occipital Lobe.Lingual Gyrus.Gray Matter.BA 17 | -2 | -94 | 4 | 3.47 | 0.0003 | 1 | 1949 |
| Right Cerebrum.Occipital Lobe.Lingual Gyrus | 12 | -78 | -4 | 3.41 | 0.0003 | 1 | 1949 |
| Right Cerebrum.Occipital Lobe.Lingual Gyrus.White Matter | 14 | -98 | -4 | 3.39 | 0.0003 | 1 | 1949 |
| Left Cerebrum.Occipital Lobe.Cuneus.White Matter | -8 | -96 | 12 | 3.25 | 0.0006 | 1 | 1949 |
| Left Cerebrum.Occipital Lobe.Cuneus.Gray Matter.BA 18 | 0 | -98 | 10 | 3.21 | 0.0007 | 1 | 1949 |

**4c. RSQ security negative correlation with Salience contrast**

| Name | X | Y | Z | Z Score | P value | Cluster ID | Cluster Size |
| --- | --- | --- | --- | --- | --- | --- | --- |
| Right Cerebrum.Temporal Lobe.Middle Temporal Gyrus.White Matter | 68 | -42 | -10 | 4.45 | 0.0000 | 1 | 1908 |
| Right Cerebrum.Temporal Lobe.Middle Temporal Gyrus.White Matter | 62 | -44 | -12 | 3.34 | 0.0004 | 1 | 1908 |
| Right Cerebrum.Temporal Lobe.Middle Temporal Gyrus.Gray Matter.BA 22 | 66 | -34 | 2 | 3.27 | 0.0005 | 1 | 1908 |
| Right Cerebrum.Temporal Lobe.Superior Temporal Gyrus.White Matter | 54 | -28 | 8 | 3.26 | 0.0006 | 1 | 1908 |
| Right Cerebrum.Parietal Lobe.Sub-Gyral.White Matter | 40 | -30 | 24 | 3.19 | 0.0007 | 1 | 1908 |
| Right Cerebrum.Limbic Lobe.Cingulate Gyrus.Gray Matter.BA 31 | 22 | -22 | 44 | 3.10 | 0.0010 | 1 | 1908 |

**4d. RSQ security negative correlation with Valence contrast**

| Name | X | Y | Z | Z Score | P value | Cluster ID | Cluster Size |
| --- | --- | --- | --- | --- | --- | --- | --- |
| Left Cerebrum.Frontal Lobe.Precentral Gyrus.White Matter | -48 | 6 | 10 | 4.12 | 0.0000 | 1 | 1772 |
| Left Cerebrum.Temporal Lobe.Middle Temporal Gyrus.Gray Matter.BA 21 | -58 | -2 | -30 | 3.64 | 0.0001 | 1 | 1772 |
| Left Cerebrum.Frontal Lobe.Inferior Frontal Gyrus.Gray Matter.BA 45 | -42 | 20 | 8 | 3.45 | 0.0003 | 1 | 1772 |
| Left Cerebrum.Frontal Lobe.Inferior Frontal Gyrus.Gray Matter.BA 47 | -54 | 20 | -2 | 3.40 | 0.0003 | 1 | 1772 |
| Left Cerebrum.Frontal Lobe.Inferior Frontal Gyrus.White Matter | -46 | 24 | -10 | 3.20 | 0.0007 | 1 | 1772 |
| Left Cerebrum.Frontal Lobe.Sub-Gyral.White Matter | -42 | 14 | 16 | 3.05 | 0.0011 | 1 | 1772 |

### Supp. Table 5: AAI vs. RSQ measured Dismissingness Brain Activity

**5a. AAI dismissingness positive correlation with Salience contrast**

| Name | X | Y | Z | Z Score | P value | Cluster ID | Cluster Size |
| --- | --- | --- | --- | --- | --- | --- | --- |
| Right Cerebrum.Occipital Lobe.Cuneus.White Matter | 14 | -92 | 12 | 2.99 | 0.0014 | 1 | 1156 |
| Right Cerebrum.Occipital Lobe.Cuneus.Gray Matter.BA 18 | 4 | -98 | 12 | 2.96 | 0.0015 | 1 | 1156 |
| Left Cerebrum.Occipital Lobe.Lingual Gyrus.Gray Matter.BA 17 | -6 | -90 | 6 | 2.81 | 0.0025 | 1 | 1156 |
| Inter-Hemispheric | 2 | -70 | 4 | 2.76 | 0.0029 | 1 | 1156 |
| Right Cerebrum.Occipital Lobe.Cuneus.White Matter | 8 | -96 | 10 | 2.70 | 0.0035 | 1 | 1156 |

**5b. AAI dismissingness negative correlation with Salience contrast**

| Name | X | Y | Z | Z Score | P value | Cluster ID | Cluster Size |
| --- | --- | --- | --- | --- | --- | --- | --- |
| Right Cerebrum.Sub-lobar.Extra-Nuclear.White Matter.Corpus Callosum | 16 | -38 | 20 | 3.59 | 0.0002 | 3 | 1779 |
| Right Cerebrum.Sub-lobar.Extra-Nuclear.White Matter.Corpus Callosum | 14 | -42 | 16 | 3.45 | 0.0003 | 3 | 1779 |
| Right Cerebrum.Sub-lobar.Extra-Nuclear.White Matter | 22 | -14 | 22 | 3.30 | 0.0005 | 3 | 1779 |
| Right Cerebrum.Sub-lobar.Extra-Nuclear.White Matter | 32 | -34 | 24 | 3.25 | 0.0006 | 3 | 1779 |
| Right Cerebrum.Sub-lobar.Extra-Nuclear.White Matter | 26 | -28 | 16 | 3.17 | 0.0008 | 3 | 1779 |
| Right Cerebrum.Limbic Lobe.Parahippocampal Gyrus | 34 | -48 | -8 | 3.11 | 0.0009 | 3 | 1779 |
| Left Cerebrum.Frontal Lobe.Sub-Gyral.White Matter | -18 | 14 | 20 | 4.14 | 0.0000 | 2 | 1230 |
| Left Cerebrum.Frontal Lobe.Medial Frontal Gyrus.White Matter | -22 | 38 | 14 | 4.01 | 0.0000 | 2 | 1230 |
| Left Cerebrum.Frontal Lobe.Sub-Gyral.White Matter | -20 | 44 | 2 | 3.47 | 0.0003 | 2 | 1230 |
| Left Cerebrum.Sub-lobar.Extra-Nuclear.White Matter | -20 | -4 | 24 | 3.15 | 0.0008 | 2 | 1230 |
| Left Cerebrum.Frontal Lobe.Sub-Gyral.White Matter | -20 | -10 | 34 | 2.76 | 0.0029 | 2 | 1230 |
| Left Cerebrum.Frontal Lobe.Sub-Gyral.White Matter | -14 | 38 | -6 | 2.60 | 0.0047 | 2 | 1230 |
| Left Cerebrum.Sub-lobar.Extra-Nuclear.White Matter | -28 | -44 | 12 | 4.19 | 0.0000 | 1 | 1172 |
| Left Cerebrum.Sub-lobar.Lateral Ventricle.Cerebro-Spinal Fluid | -30 | -46 | 4 | 3.89 | 0.0001 | 1 | 1172 |
| Left Cerebrum.Temporal Lobe.Sub-Gyral.White Matter | -36 | -54 | 0 | 3.87 | 0.0001 | 1 | 1172 |
| Left Cerebrum.Sub-lobar.Extra-Nuclear.White Matter | -18 | -50 | 14 | 3.24 | 0.0006 | 1 | 1172 |
| Left Cerebrum.Temporal Lobe.Sub-Gyral.White Matter | -26 | -56 | 16 | 2.89 | 0.0019 | 1 | 1172 |
| Left Cerebrum.Sub-lobar.Extra-Nuclear.White Matter | -26 | -62 | 12 | 2.81 | 0.0025 | 1 | 1172 |

**5c. RSQ dismissingness negative correlation with Salience contrast**

| Name | X | Y | Z | Z Score | P value | Cluster ID | Cluster Size |
| --- | --- | --- | --- | --- | --- | --- | --- |
| Right Cerebellum.Posterior Lobe.Declive.Gray Matter | 14 | -76 | -22 | 5.19 | 0.0000 | 3 | 2712 |
| Left Cerebellum.Posterior Lobe.Uvula.Gray Matter | -8 | -68 | -32 | 4.09 | 0.0000 | 3 | 2712 |
| Left Cerebellum.Posterior Lobe.Tuber.Gray Matter | -48 | -70 | -28 | 3.96 | 0.0000 | 3 | 2712 |
| Left Cerebellum.Posterior Lobe.Pyramis.Gray Matter | -48 | -74 | -36 | 3.95 | 0.0000 | 3 | 2712 |
| Left Cerebellum.Posterior Lobe.Pyramis.Gray Matter | -36 | -80 | -32 | 3.90 | 0.0000 | 3 | 2712 |
| Right Cerebrum.Occipital Lobe.Cuneus.White Matter | 12 | -98 | 16 | 3.88 | 0.0001 | 3 | 2712 |
| Right Cerebrum.Limbic Lobe.Cingulate Gyrus.White Matter | 14 | -2 | 36 | 4.28 | 0.0000 | 2 | 2540 |
| Right Cerebrum.Frontal Lobe.Precentral Gyrus.White Matter | 46 | -8 | 42 | 4.13 | 0.0000 | 2 | 2540 |
| Right Cerebrum.Limbic Lobe.Cingulate Gyrus.Gray Matter.BA 24 | 6 | 0 | 42 | 3.79 | 0.0001 | 2 | 2540 |
| Right Cerebrum.Parietal Lobe.Postcentral Gyrus.White Matter | 50 | -18 | 40 | 3.71 | 0.0001 | 2 | 2540 |
| Right Cerebrum.Sub-lobar.Extra-Nuclear.White Matter.Corpus Callosum | 12 | -2 | 24 | 3.61 | 0.0002 | 2 | 2540 |
| Right Cerebrum.Parietal Lobe.Inferior Parietal Lobule.White Matter | 54 | -36 | 42 | 3.41 | 0.0003 | 2 | 2540 |
| Left Cerebrum.Temporal Lobe.Superior Temporal Gyrus.Gray Matter.BA 22 | -48 | 6 | -8 | 3.41 | 0.0003 | 1 | 1494 |
| Left Cerebrum.Temporal Lobe.Superior Temporal Gyrus.White Matter | -56 | -30 | 8 | 3.32 | 0.0005 | 1 | 1494 |
| Left Cerebrum.Frontal Lobe.Precentral Gyrus.Gray Matter.BA 6 | -64 | 4 | 4 | 3.13 | 0.0009 | 1 | 1494 |
| Left Cerebrum.Temporal Lobe.Superior Temporal Gyrus.Gray Matter.BA 38 | -58 | 12 | -12 | 3.13 | 0.0009 | 1 | 1494 |
| Left Cerebrum.Sub-lobar.Extra-Nuclear.White Matter | -42 | -16 | 26 | 3.12 | 0.0009 | 1 | 1494 |

**5d. AAI dismissingness positive correlation with Valence contrast**

| Name | X | Y | Z | Z Score | P value | Cluster ID | Cluster Size |
| --- | --- | --- | --- | --- | --- | --- | --- |
| Left Cerebrum.Sub-lobar.Thalamus.Gray Matter | -8 | -22 | 0 | 4.42 | 0.0000 | 2 | 2285 |
| Right Cerebrum.Sub-lobar.Thalamus.Gray Matter.Mammillary Body | 12 | -18 | 2 | 4.02 | 0.0000 | 2 | 2285 |
| Left Cerebrum.Parietal Lobe.Inferior Parietal Lobule.White Matter | -42 | -32 | 42 | 3.34 | 0.0004 | 2 | 2285 |
| Left Cerebrum.Parietal Lobe.Postcentral Gyrus.White Matter | -48 | -20 | 24 | 3.06 | 0.0011 | 2 | 2285 |
| Left Cerebrum.Parietal Lobe.Supramarginal Gyrus.White Matter | -52 | -36 | 32 | 3.06 | 0.0011 | 2 | 2285 |
| Left Cerebrum.Temporal Lobe.Superior Temporal Gyrus.Gray Matter.BA 41 | -48 | -32 | 12 | 2.99 | 0.0014 | 2 | 2285 |
| Right Cerebrum.Occipital Lobe.Lingual Gyrus.Gray Matter.BA 18 | 12 | -80 | -2 | 4.08 | 0.0000 | 1 | 2174 |
| Left Cerebrum.Occipital Lobe.Lingual Gyrus | -4 | -76 | -2 | 3.99 | 0.0000 | 1 | 2174 |
| Right Cerebrum.Occipital Lobe.Cuneus.Gray Matter.BA 18 | 4 | -98 | 12 | 3.79 | 0.0001 | 1 | 2174 |
| Right Cerebrum.Occipital Lobe.Middle Occipital Gyrus.Gray Matter.BA 18 | 14 | -96 | 20 | 3.51 | 0.0002 | 1 | 2174 |
| Left Cerebrum.Occipital Lobe.Lingual Gyrus.Gray Matter.BA 17 | -2 | -100 | 2 | 3.35 | 0.0004 | 1 | 2174 |
| Right Cerebrum.Occipital Lobe | 4 | -76 | 8 | 3.09 | 0.0010 | 1 | 2174 |

**5e. AAI dismissingness negative correlation with Valence contrast**

| Name | X | Y | Z | Z Score | P value | Cluster ID | Cluster Size |
| --- | --- | --- | --- | --- | --- | --- | --- |
| Right Cerebrum.Sub-lobar.Extra-Nuclear.White Matter.Corpus Callosum | 18 | -40 | 14 | 3.85 | 0.0001 | 2 | 2336 |
| Right Cerebrum.Temporal Lobe.Sub-Gyral.White Matter | 36 | -50 | 16 | 3.73 | 0.0001 | 2 | 2336 |
| Right Cerebrum.Sub-lobar.Extra-Nuclear.White Matter | 24 | -32 | 22 | 3.66 | 0.0001 | 2 | 2336 |
| Right Cerebrum.Sub-lobar.Extra-Nuclear.White Matter | 16 | -40 | 24 | 3.55 | 0.0002 | 2 | 2336 |
| Right Cerebrum.Limbic Lobe.Cingulate Gyrus.White Matter | 18 | -12 | 26 | 2.91 | 0.0018 | 2 | 2336 |
| Right Cerebrum.Sub-lobar.Extra-Nuclear.White Matter | 28 | -28 | 12 | 2.88 | 0.0020 | 2 | 2336 |
| Left Cerebrum.Temporal Lobe.Sub-Gyral.White Matter | -26 | -42 | 2 | 4.00 | 0.0000 | 1 | 2257 |
| Left Cerebrum.Sub-lobar.Extra-Nuclear.White Matter.Corpus Callosum | -14 | -42 | 16 | 3.93 | 0.0000 | 1 | 2257 |
| Left Cerebrum.Temporal Lobe.Sub-Gyral.White Matter | -32 | -56 | -2 | 3.92 | 0.0000 | 1 | 2257 |
| Left Cerebrum.Sub-lobar.Extra-Nuclear.White Matter.Corpus Callosum | -18 | -48 | 16 | 3.79 | 0.0001 | 1 | 2257 |
| Left Cerebrum.Frontal Lobe.Sub-Gyral.White Matter | -22 | -12 | 38 | 3.49 | 0.0002 | 1 | 2257 |
| Left Cerebrum.Sub-lobar.Extra-Nuclear.White Matter | -20 | -20 | 22 | 3.44 | 0.0003 | 1 | 2257 |

### Supp. Table 6: AAI vs. RSQ Interactions with Mood (BAI+BAI)

| 6a. AAI security positive interaction with mood in predicting Salience contrast Activity | | | | | | | |
| --- | --- | --- | --- | --- | --- | --- | --- |
| Name | X | Y | Z | Z score | P value | ClusterID | ClusterSize |
| Right Temporal Lobe Subcortical White Matter | 34 | -58 | 12 | 3.94 | 0.00023 | 3 | 952 |
| Right Caudate Tail | 26 | -36 | 10 | 5.6 | 0.00447 | 2 | 616 |

| 6b. AAI security negative interaction with mood in predicting Salience contrast Activity | | | | | | | |
| --- | --- | --- | --- | --- | --- | --- | --- |
| Name | X | Y | Z | Z score | P value | ClusterID | ClusterSize |
| Right BA 18—Occipital Lobe, Cuneus | 10 | -70 | 20 | 4.88 | <0.00001 | 2 | 1493 |
| Right BA 8—Superior Frontal Gyrus | 16 | 46 | 52 | 4.34 | 0.00395 | 1 | 629 |

| 6c. AAI security negative interaction with mood in predicting Valence contrast Activity | | | | | | | |
| --- | --- | --- | --- | --- | --- | --- | --- |
| Name | X | Y | Z | Z score | P value | ClusterID | ClusterSize |
| Right BA 21—Middle Temporal Gyrus | 62 | -16 | -2 | 5.21 | 0.000253 | 2 | 938 |

| 6d. AAI dismissiveness negative interaction with mood in predicting Salience contrast Activity | | | | | | | | | |
| --- | --- | --- | --- | --- | --- | --- | --- | --- | --- |
| Name | X | Y | Z | Z score | | P value | ClusterID | | ClusterSize |
| Right BA 19–V3 Visual Cortex | 4 | -96 | 28 | | 5.52 | <0.00001 | 3 | 2996 | |
| Right BA 32–Medial Frontal Gyrus | 18 | 14 | 42 | | 4.88 | 0.00107 | 2 | 771 | |

| 6e. AAI dismissiveness negative interaction with mood in predicting Valence contrast Activity | | | | | | | | |
| --- | --- | --- | --- | --- | --- | --- | --- | --- |
| Name | X | Y | Z | Z score | P value | ClusterID | | ClusterSize |
| Right BA 7–Somatosensory Association Cortex (precuneus) | 20 | -88 | 50 | 5.31 | <0.00001 | 3 | 1776 | |
| Left BA 5–Somatosensory Association Cortex | 0 | -44 | 64 | 5.81 | 0.00001 | 2 | 1317 | |

| 6f. RSQ secure-fearful negative interaction with mood in predicting Salience contrast Activity | | | | | | | |
| --- | --- | --- | --- | --- | --- | --- | --- |
| Name | X | Y | Z | Z score | P value | ClusterID | ClusterSize |
| Right BA 7–Somatosensory Association Cortex (precuneus) | 10 | -88 | 50 | 6.70 | <0.00001 | 4 | 1591 |
| Right BA 21–Middle Temporal gyrus | 68 | -10 | -6 | 5.32 | <0.00001 | 3 | 1362 |
| Left BA 21–Middle Temporal gyrus | -72 | -22 | -6 | 5.41 | 0.00021 | 2 | 959 |

| 6g. RSQ secure-fearful positive interaction with mood in predicting Valence contrast Activity | | | | | | | |
| --- | --- | --- | --- | --- | --- | --- | --- |
| Name | X | Y | Z | Z score | P value | ClusterID | ClusterSize |
| Left BA 40–Supramarginal gyrus (Wernicke's area) | -48 | -44 | 54 | 5.33 | <0.00001 | 1 | 1561 |

| 6h. RSQ secure-fearful negative interaction with mood in predicting Valence contrast Activity | | | | | | | |
| --- | --- | --- | --- | --- | --- | --- | --- |
| Name | X | Y | Z | Z score | P value | ClusterID | ClusterSize |
| Left BA 19–V3 Visual Cortex | -26 | -100 | 32 | 5.54 | 0.00215 | 3 | 694 |

| 6i. RSQ dismissing-preoccupied positive interaction with mood in predicting Salience contrast Activity | | | | | | | | |
| --- | --- | --- | --- | --- | --- | --- | --- | --- |
| Name | | X | Y | Z | Z score | P value | ClusterID | ClusterSize |
| Right BA 6–Premotor And Supplementary Motor Cortex | 8 | | -14 | 72 | 7.62 | 0.00007 | 2 | 1098 |

| 6j. RSQ dismissing-preoccupied negative interaction with mood in predicting Salience contrast Activity | | | | | | | |
| --- | --- | --- | --- | --- | --- | --- | --- |
| Name | X | Y | Z | Z score | P value | ClusterID | ClusterSize |
| Right BA 10–Frontopolar Area | 2 | 70 | -6 | 6.02 | <0.00001 | 3 | 1613 |
| Right BA 7–Somatosensory Association Cortex (precuneus) | 2 | -58 | 76 | 6.28 | 0.00022 | 2 | 956 |
| Left BA 2–Primary Somatosensory Cortex | -34 | -38 | 62 | 5.41 | 0.00366 | 1 | 637 |

| 6k. RSQ dismissing-preoccupied negative interaction with mood in predicting Valence contrast Activity | | | | | | | |
| --- | --- | --- | --- | --- | --- | --- | --- |
| Name | X | Y | Z | Z score | P value | ClusterID | ClusterSize |
| Left BA 7 - Somatosensory Association Cortex | -38 | -36 | 72 | 7.60 | 0.00017 | 1 | 989 |
