# Supplementary figures and images for: Comparison of Brain Activity Correlating with Self-Report versus Narrative Attachment Measures during Conscious Appraisal of an Attachment Figure
Source: Front Hum Neurosci. 2016 Mar 14;10:90. doi: 10.3389/fnhum.2016.00090 (PMC4789543; doi:10.3389/fnhum.2016.00090)

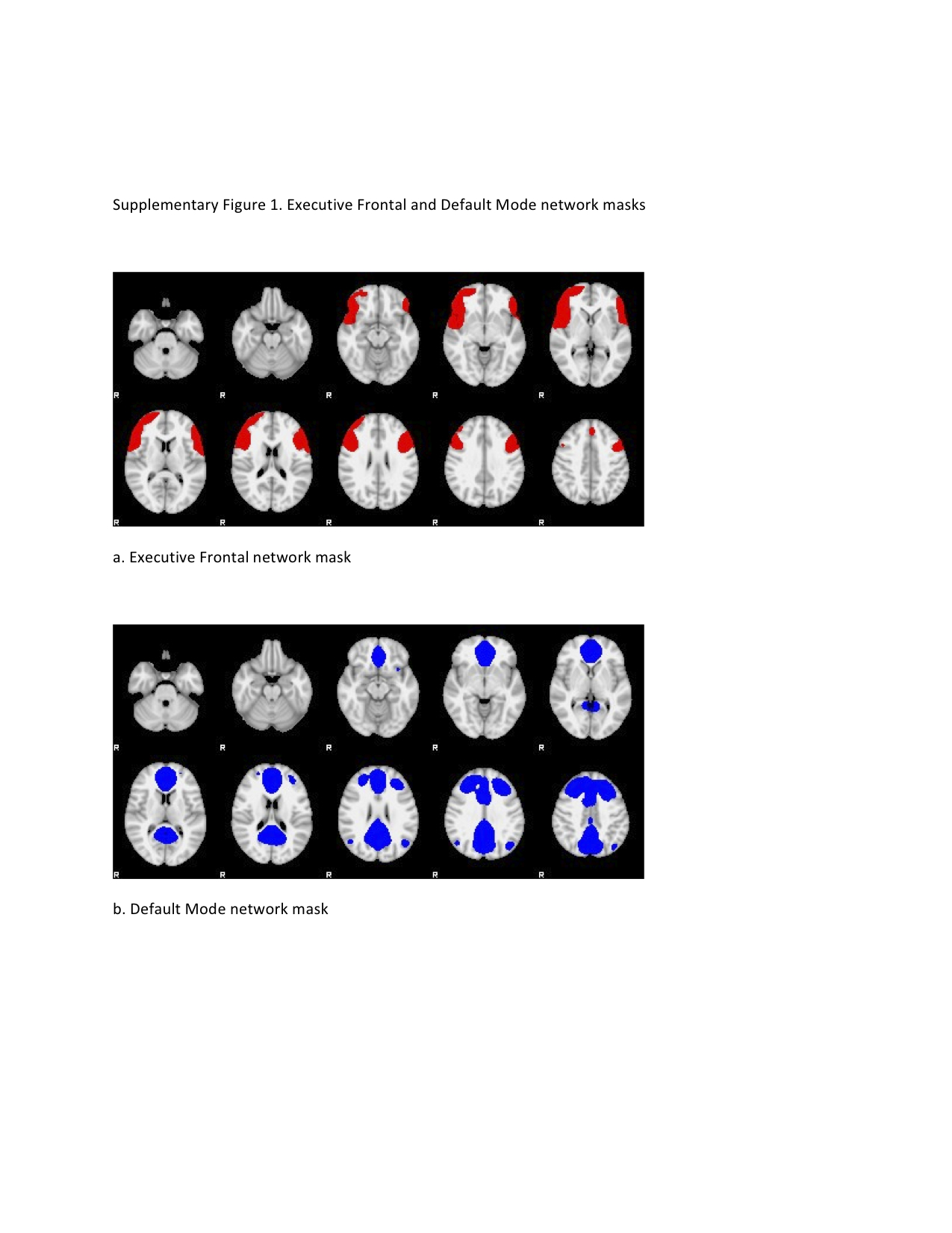

Supplement: Supplementary file 2 [file Image_1.JPEG]
