# Supplementary material for: Comparison of Brain Activity Correlating with Self-Report versus Narrative Attachment Measures during Conscious Appraisal of an Attachment Figure
Source: Front Hum Neurosci. 2016 Mar 14;10:90. doi: 10.3389/fnhum.2016.00090 (PMC4789543; doi:10.3389/fnhum.2016.00090)
Supplement: Supplementary file 3 [file Image_2.PDF]

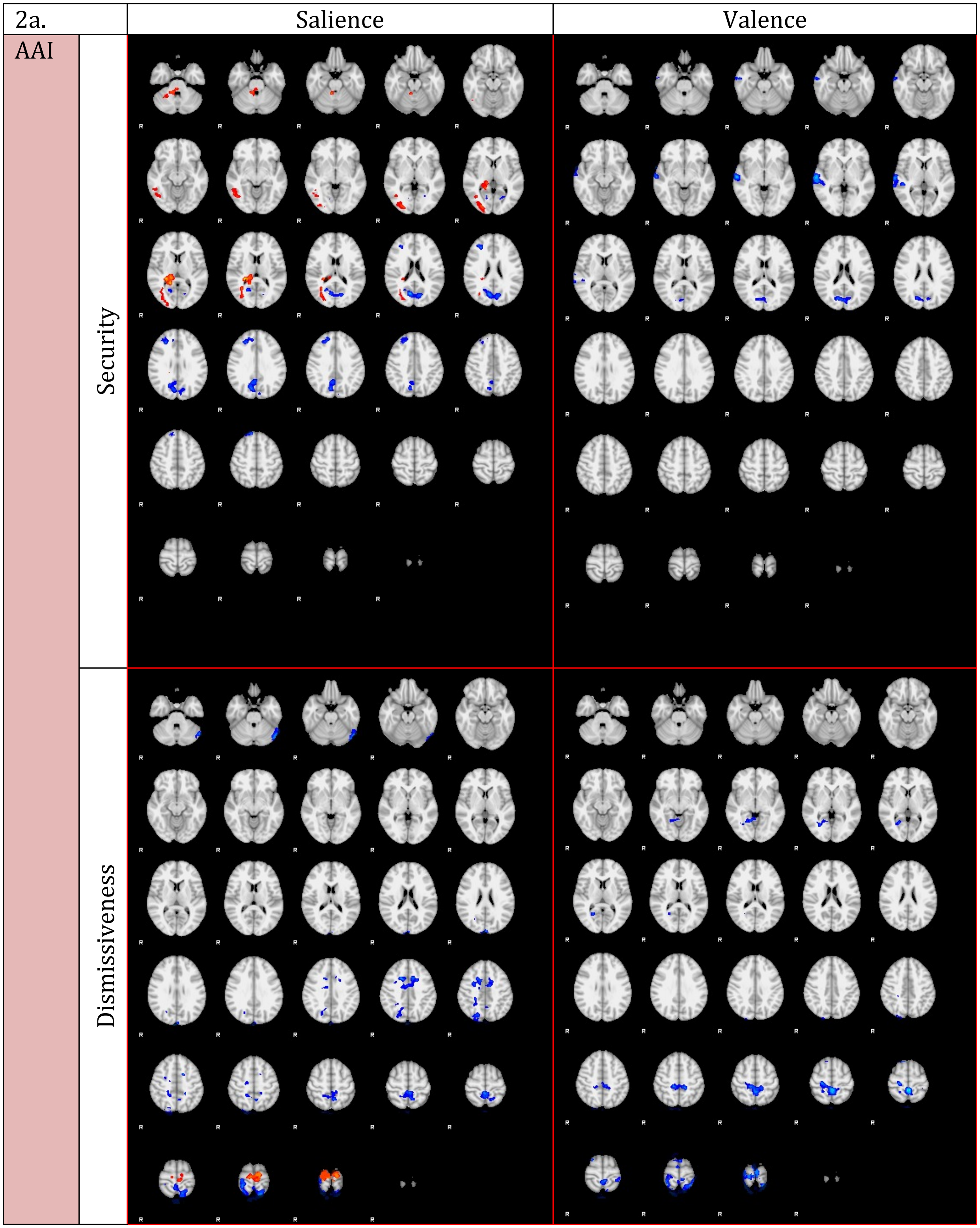
2a.
Salience
Valence
AAI
Security
Dismissiveness

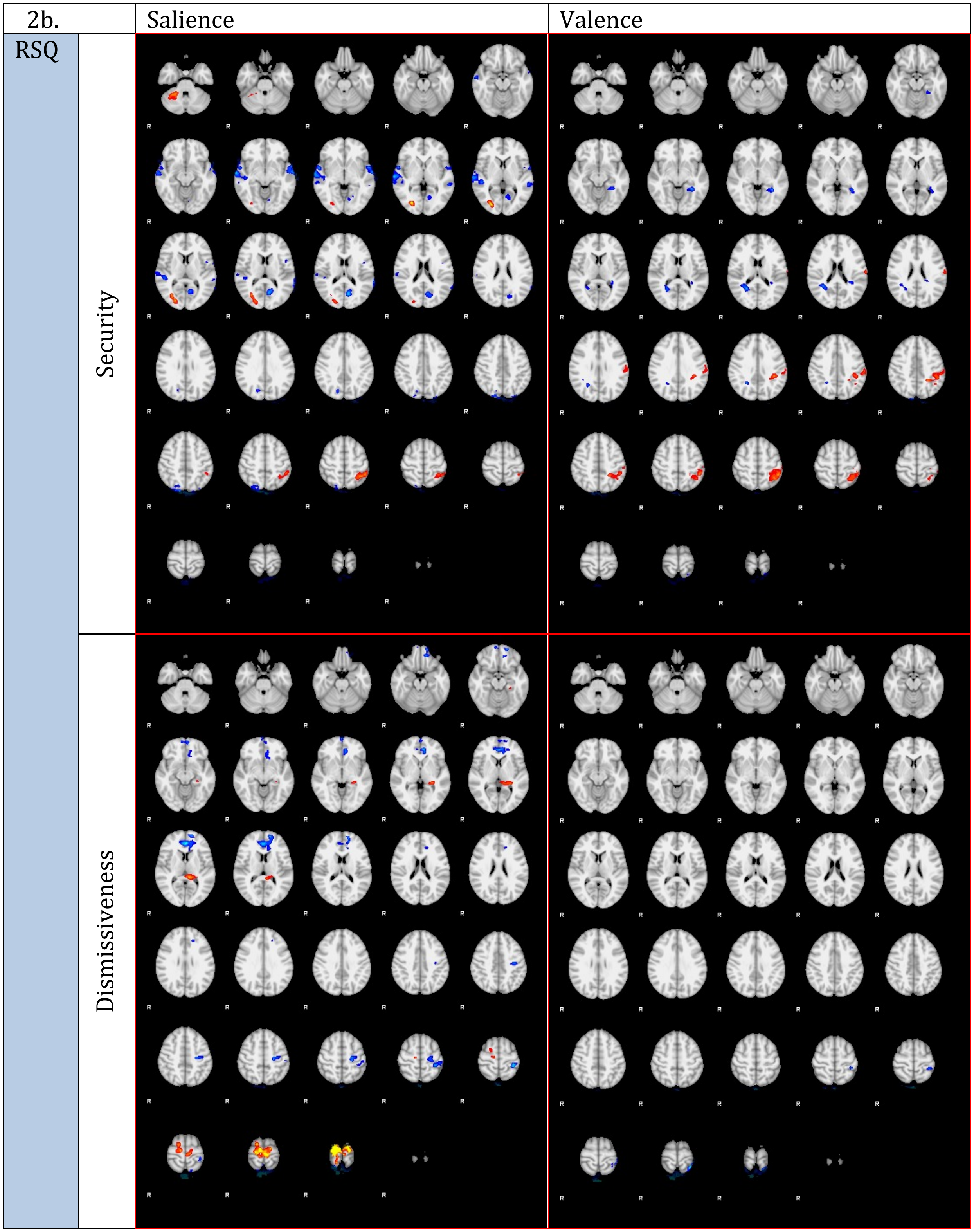

Supplementary Figure 2. Regions of significant interaction between Mood and Attachment measures.

2a. Interactions for AAI measures. 2b. Interactions for RSQ measures.

Red regions represent significant positive interaction blue regions represent significant negative interaction.
